# Modeling of Thermo-Chemo-Mechanical Properties of Anode Mixture during the Baking Process

**DOI:** 10.3390/ma14154320

**Published:** 2021-08-02

**Authors:** Bowen Chen, Hicham Chaouki, Donald Picard, Julien Lauzon-Gauthier, Houshang Alamdari, Mario Fafard

**Affiliations:** 1 Department of Civil and Water Engineering, NSERC/Alcoa Industrial Research Chair MACE3, Aluminium Research Centre—REGAL, Université Laval, Quebec, QC G1V 0A6, Canada; hicham.chaouki@gci.ulaval.ca (H.C.); mario.fafard.2@ulaval.ca (M.F.); 2 Eddify Technologies Company, Quebec, QC G1P 0B3, Canada; dpicard@eddyfi.com; 3 Continuous Improvement Smelting Technology, Alcoa, Deschambault-Grondines, QC G0A 1S0, Canada; julien.lauzon-gauthier@alcoa.com; 4 Department of Mining, Metallurgical and Materials Engineering, NSERC/Alcoa Industrial Research Chair MACE3, Aluminium Research Centre—REGAL, Université Laval, Quebec, QC G1V 0A6, Canada; houshang.alamdari@gmn.ulaval.ca

**Keywords:** Hall–Héroult process, prebaked anode, thermogravimetric analysis, dilatometry, creep test, shrinking index

## Abstract

In the Hall–Héroult process, prebaked carbon anodes are utilized to produce primary aluminium. The quality of the anode plays a crucial role in the efficiency of electrowinning primary aluminium. In the production of anodes, the anode baking is considered as the stage most frequently causing anode problems. During the baking process, the anode undergoes complex physicochemical transformations. Moreover, the anode at a lower position, imposed by loading pressures from upper anodes, will creep during this process. Thus, the production of high-quality anodes demands efficient control of their baking process. This paper aims to investigate the thermo-chemo-mechanical properties of the anode paste mixture at high temperatures. These properties include kinetic parameters of pitch pyrolysis such as the activation energy and the pre-exponential factor, the thermal expansion coefficient (TEC) and relevant mechanical parameters related to the elastic, the viscoelastic and the viscoplastic behaviours of the anode. For this purpose, experiments consisting of the thermogravimetric analysis, the dilatometry and the creep test were carried out. Based on the obtained results, the forementioned parameters were identified. Relevant mechanical parameters were expressed as a function of a new variable, called the shrinking index, which is related to the volatile released in open and closed pores of the anode. This variable would be used to highlight the chemo-mechanical coupling effect of the anode mixture. New insights into the phenomena such as the expansion due to the increase of the pore pressure and the chemical shrinkage of the anode during the baking process were also gained in this work. These investigations pave the way for modeling the thermo-chemo-poromechanical behaviour of the anode during the baking process.

## 1. Introduction

In the Hall–Héroult electrolysis process, prebaked carbon anodes are utilized to produce primary aluminium. High-quality anodes are required to increase the efficiency of the electrolysis process [1]. In the anode production, the anode baking is considered as the stage most frequently causing anode problems such as low mechanical strength and thermal shock resistance of the anode [2]. Green anodes are fabricated by vibro-compaction/compaction method [3]. They typically consist of petroleum coke aggregates (≈65 wt %), coal tar pitch (≈15 wt %) and recycled anode butts (≈20 wt %) [4]. During the baking process, green anodes are stacked in pits in a refractory furnace [5]. The anodes in pits suffer from the thermal expansion, the expansion due to the entrapment of the released volatile and the chemical shrinkage [6]. It is known that the thermal stress causing the thermal expansion and the chemical shrinkage during the baking process can lead to the anode cracking which decreases the mechanical strength and the thermal shock resistance of the anode [2,6]. Devolatilization of the pitch can crack the anode by increasing the internal gas pressure if a high heating rate is used [7]. Furthermore, imposed by the loading pressure from upper anodes, the anode at a lower position will creep, which causes a permanent deformation of the anode [8]. Thus, thermo-chemo-mechanical properties of the anode will be greatly changed during the baking process.

To study the thermo-chemical effect of the baking anode, kinetics of pitch pyrolysis should be quantitatively analyzed. In [9,10,11], the thermogravimetric analysis (TGA) was used to investigate the chemical pyrolysis of the carbon electrodes and kinetic parameters in modeling the pitch pyrolysis process such as the activation energy and pre-exponential factor were identified using a power-law model. In [12], different methods of identifying these kinetic parameters were further proposed and discussed.

The swelling and the shrinkage of the anode associated with the pitch pyrolysis occur at high temperatures, i.e., from 200 to 600 °C [13]. The swelling of the anode is a combination of the thermal expansion and the expansion due to the increase in the pore pressure caused by the entrapped volatile. In [14], the expansion of a carbon material, due to the increase in the pore pressure created by the entrapped air and low-boiling fractions of the pitch, was observed in the temperature range of 150 to 250 °C using a heating rate of 4 °C/min. In [8], the expansion of the anode associated with the pitch pyrolysis was observed starting from 250 °C up to around 480 °C at a heating rate of 11 °C/h. Nevertheless, few studies have been done to investigate the thermal expansion and the thermal expansion coefficient (TEC) evolution of the carbon anode as a function of the temperature. In [15], the thermal expansion coefficient (TEC) of a carbon mixture was experimentally found to decrease from 500 to 1000 °C.

The shrinkage behaviour was found to exist in carbon materials at high temperatures. When the pitch binder begins to transform into coke, the carbon material begins to shrink [15]. On the microscopic level, the shrinkage of the carbon material was caused by the shrinking of binder coke bridges in the pitch [16,17,18]. Macroscopically, dilatometry performed by [14,19] has demonstrated that the pitch-bonded carbons exhibit a shrinkage during the baking process. Such a behaviour was further investigated in the anode material [6], which was observed after around 530 °C and before reaching 950 °C, during which time the chemical shrinkage overwhelmed the thermal expansion.

Furthermore, carbon materials were found to creep at high temperatures mainly due to the deformation of the binder bridges [16]. Because of the rheological ability of the coal tar pitch [20] and the temperature dependency of viscoelastic properties of coke-pitch disperse system at low temperatures under 180 °C [21], the carbon anodes are supposed to have a time-dependent behaviour under loading below 200 °C. Moreover, in [22], before being carbonized into a solid green coke at 627 °C, the pitch was transformed into a mesophase having a structure that can be deformed by mechanical agencies with limited possibility for recovery. Thus, the carbon anodes are expected to creep above 200 °C. In [8], the carbon anodes demonstrated a creep behaviour below 500 °C at different baking temperatures, which is due to the change in the pitch viscosity with respect to the temperature. However, the modeling of the creep behaviour of the carbon anodes at high temperatures still remains not well-understood.

Time-dependent behaviours were also found in other carbon materials used in the Hall–Héroult process such as the carbon cathode [23] and the ramming paste [24]. Some rheological models have been proposed for modeling their creep behaviours. In [25], the viscoelastic behaviour of the carbon cathode was modeled at the room temperature. In [26], the viscoelastic–viscoplastic behaviour of the ramming paste was modelled at high temperatures. Nevertheless, they did not consider the impact of the pitch pyrolysis on the mechanical properties of the carbon materials during the baking process. In [27], a thermo-chemo-poromechanical model for the baking anode, founded on the thermodynamic framework of poromechanics, was proposed. In this model, a new internal variable called the “shrinking index”, representing the chemical reaction of pitch pyrolysis, was introduced to take into account the chemo-mechanical coupling effect.

This paper aims to investigate the following properties of the anode during the baking process: (i) kinetic parameters of pitch pyrolysis such as the activation energy and pre-exponential factor; (ii) the thermal expansion coefficient (TEC) and (iii) mechanical parameters related to the elastic, the viscoelastic and the viscoplastic behaviours of the anode. For this purpose, experimental characterizations were carried out: (i) the thermogravimetric analysis (TGA) to obtain the evolution of the anode mass; (ii) the dilatometry to obtain the thermal and the chemical strains and (iii) the creep test to obtain the creep strain under a constant loading at high temperatures. To this end, lab-scale anode samples were fabricated using both the servohydraulic compaction method [28] and the Proctor compaction method [29]. In an industrial baking furnace, observations in [30] showed that, an increase in the heating rate from 10 to 15 °C/h would increase the activation energy, the pre-exponential factor, and the internal gas pressure of the anode. Since this work aims to highlight the effect of the pitch pyrolysis reaction on the thermal-mechanical behaviour of the anode, a heating rate of 20 °C/h was used in the temperature range from 150 to 650 °C where the most complex physico-chemical phenomena takes place. Based on the obtained results, parameters involved in the power-law model were identified [9,12]. Thermal expansion coefficient (TEC) was calculated using the cooling period of the strain in the dilatometry. Concerning the mechanical behaviour, Burger’s rheological model was used for modeling the creep behaviour of the anode at high temperatures. The stress-strain relation corresponding to the viscoelastic behaviour of a carbon cathode, developed in [25], was used. Mechanical parameters involved in the Burger’s rheological model were inversely identified such that the viscoelastic and viscoplastic strains in the axial direction at high temperatures were, respectively, predicted. Relevant identified parameters were further expressed as a function of the shrinking index.

The motivation of this work was to characterize thermo-chemo-mechanical properties of the anode mixture at high temperatures and to highlight the chemo-mechanical coupling effect of the anode mixture using the shrinking index. Characterized physical and mechanical properties would provide an outlet for modeling the thermo-chemo-poromechanical behaviour of the anode mixture during the baking process.

## 2. Methodology

### 2.1. Preparation of Anode Samples

The raw materials of the anode paste, used to fabricate laboratory-scale green anode samples, were provided by Alcoa Deschambault (Deschambault, QC, Canada). The recipe is composed of the calcined coke aggregates (86.06 wt %) (≤8 US Mesh) and the coal tar pitch (13.94 wt %). The size distribution of coke aggregates in the anode sample is listed in Table 1.

**Figure 1:**
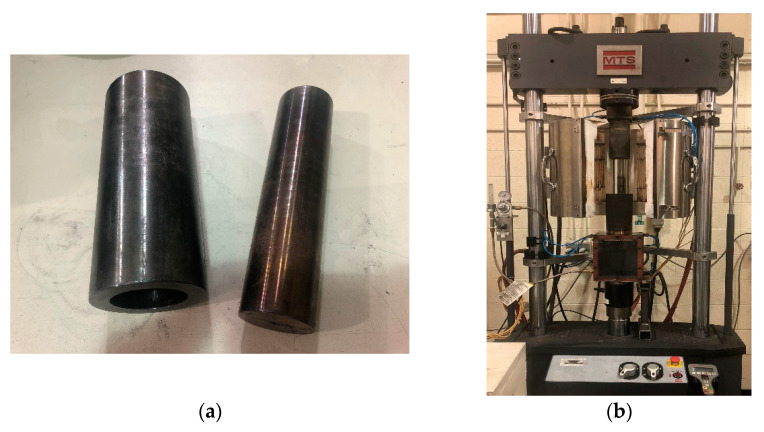
Device for the compaction of green anode samples by MTS servohydraulic press: (**a**) rigid die; (**b**) MTS servohydraulic press.

The green anode samples, with dimensions 50 × 100 mm (diameter × height), were fabricated using a MTS Servohydraulic press (MTS, Eden Prairie, MN, USA) as shown in Figure 1. The compaction of the anode paste was performed at 150 °C under a maximum uniaxial pressure of 70 MPa [28]. The samples were cut into ones having dimensions 50 × 50 mm (diameter × height), which were used for the thermogravimetric analysis (TGA) and the dilatometry.

**Figure 2:**
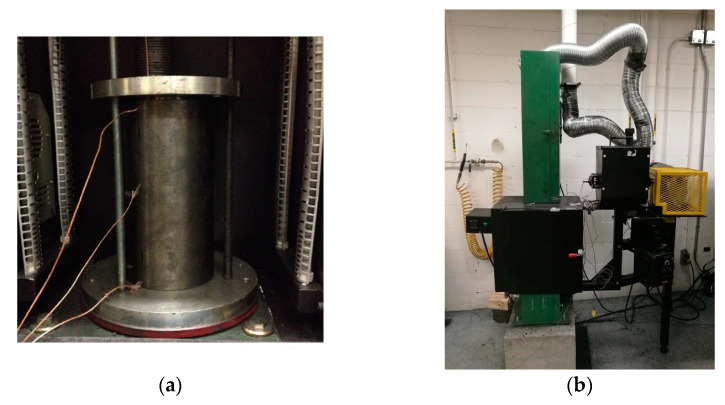
Device for the compaction of green anode samples by Proctor: (**a**) rotation mold; (**b**) Proctor.

The green anode samples, with dimensions 100 × 200 mm (diameter × height), were fabricated using a Proctor as shown in Figure 2. The compaction was performed in a rotation mold in the Proctor at the operation temperature of 150 °C [29]. The samples were drilled and cut into small ones having dimensions 50 × 100 mm (diameter × height), which were used for the creep test.

**Figure 3:**
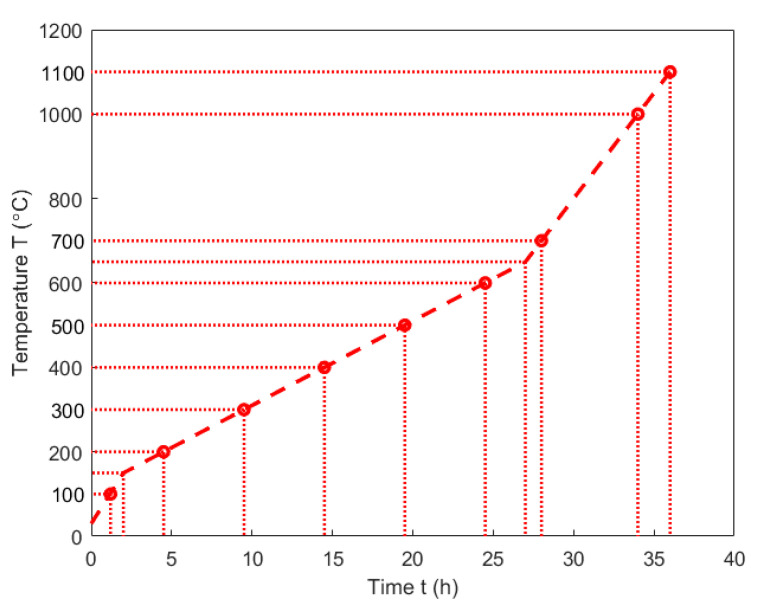
Heating program [31].

The heating program, as shown in Figure 3, was used for the TGA analysis, the dilatometry and the creep test. Red dashed line corresponds to the heating program of the TGA analysis, where the samples were baked up to 1000 °C. Red circles correspond to given target temperatures $T^{*}$. Samples were baked up to $T^{*}$ and cooled down to the room temperature for the dilatometry ($T^{*}$ ranging from 100 to 1100 °C excluding 1000 °C) and for the creep test ($T^{*}$ ranging from 200 to 700 °C). Such a baking of samples, for the creep test, aims to eliminate the volatile content in open pores, which is generated by the chemical reaction of pitch pyrolysis. Samples aimed for different characterizations are categorized in Table 2.

### 2.2. Thermogravimetric Analysis (TGA)

**Figure 4:**
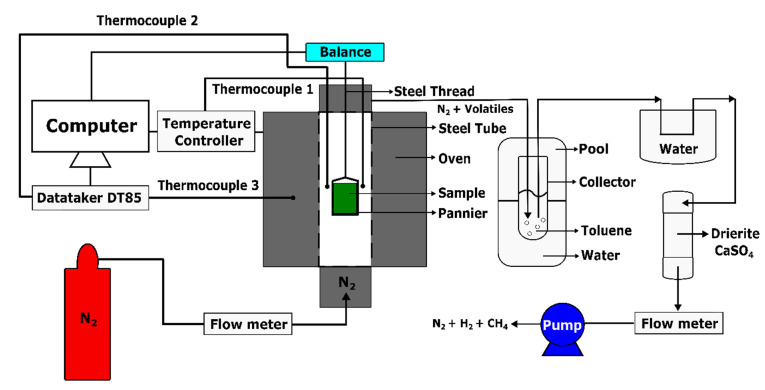
Schematic representation of TGA set-up.

The thermogravimetric analysis was intended to obtain the evolution of sample mass as a function of the temperature for studying the kinetic behaviour of the pitch pyrolysis. This analysis was carried out using an experimental set-up, as shown in Figure 4. This set-up was developed in the Aluminium Research Centre—REGAL at Laval University (Québec, QC, Canada), according to the International Standard ISO 12988-2 [32]. It consists of a baking oven (ATS: Applied Test System) that can reach a maximum temperature of 1200 °C. The oven contains a steel tube used as a pyrolysis chamber. In the tube, the green sample was held by a pannier connected to an analytical balance (Mettler Toledo ML802T, Columbus, OH, USA) having a precision of 0.01 g, through a steel thread. The thermocouples 1 and 2 were located in the steel tube. They were, respectively, used to control the oven temperature and to measure the sample temperature. Thermocouple 3 was located in the oven to observe the oven temperature. The pipe, installed at the top of the tube, was used to conduct out the exhausted gas. To avoid the sample oxidation during the baking process, the bottom of the steel tube was connected to a support that supplies the pyrolysis chamber with compressed nitrogen (N_2_) at a flow rate of 2 L/min.

During the heating period, the released volatile mixed up with the nitrogen and passed into a glass collector, which was used to dissolve the condensable part of the volatile in the toluene. To fully eliminate the condensable volatile, it was further condensed in a cold-water environment and conducted through the drierite (CaSO_4_). Finally, the non-condensable volatile with the nitrogen was pumped out at a flow rate of 1.5 L/min. The frequency of the data measurement was 0.2 Hz, and the data were integrated using a LabView software and an acquisition center (DataTaker DT85).

### 2.3. Dilatometry

#### 2.3.1. Device Description and Experimental Procedure

**Figure 5:**
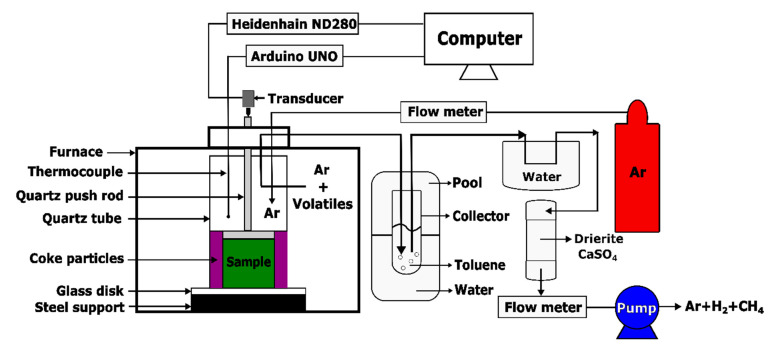
Schematic representation of dilatometry set-up.

The dilatometry test aims to characterize displacements of the anode in the axial direction for different target temperatures. The results are used to calculate the thermal expansion coefficient in the Section 3.2.1. The dilatometry was performed using an experimental set-up, as shown in Figure 5. This set-up was established in the Aluminium Research Centre—REGAL at Laval University, according to the International Standard ISO 14428 [33]. It is composed of a baking furnace (ATS Series 3150 Front Load Box Furnace (ATS, Butler, PA, USA) that can bake the sample up to different target temperatures under 1200 °C. The anode sample was placed in a quartz tube located at the center of the furnace. During the baking, the vertical displacement of the sample was transmitted to the transducer through a light-weight quartz push-rod. The transducer was connected to a Heidenhain (ND280), which was used to measure the displacement with required precision. A thermocouple was placed near the sample to measure its temperature. An Arduino UNO, connected to the thermocouple, served to record the data. At the top of the furnace, a pipe ventilated out the exhausted gas. To avoid the sample oxidation during the baking, the anode sample was surrounded by coke particles (−30 + 50 US mesh) and the argon was injected into the quartz tube at a flow rate of 2 L/min.

During the heating period (Figure 3), the released volatile mixed up with the argon and passed into a condensation system consisting of toluene, cold water environment and drierite (CaSO_4_), as explained in Section 2.2. Finally, the non-condensable volatile with the argon was exhausted out by a vacuum pump at a flow rate of 1.5 L/min. The data were recorded at a frequency of 0.2 Hz and integrated using a MATLAB programming system (Appdesigner).

#### 2.3.2. Characterization Procedure

The strain of the sample in the axial direction was obtained by characterizing the axial displacement of the sample. However, the components in the apparatus such as the quartz push-rod, the glass disk and the steel support suffer from a thermal expansion which overlaps the sample displacement. To distinguish the displacement of the sample, the total displacement measured by the transducer should be corrected by extracting the displacement caused by the thermal expansion of components in the apparatus as follows:

(1) $$\Delta h_{t}^{\mathrm{tot}}=\Delta h_{t}^{D}-\Delta h_{t}^{s}$$

where $\Delta h_{t}^{\mathrm{tot}}$ is the total displacement of the sample in the axial direction at a given time t; $\Delta h_{t}^{D}$ is the total displacement measured by the transducer and $\Delta h_{t}^{s}$ is the displacement caused by the thermal expansion of components in the apparatus.

To characterize $\Delta h_{t}^{s}$, the equipment was calibrated using a fused quartz glass as the reference material with the same dimensions as the anode sample and a thermal expansion coefficient equal to $\alpha_{q}=5.5\times 10^{-7}\;{}^{\circ}C^{-1}$. It is calculated as follows:

(2) $$\Delta h_{t}^{s}=\Delta h_{t}^{\mathrm{ct}}-\Delta h_{t}^{q}$$

where $\Delta h_{t}^{\mathrm{ct}}$ is the calibrated displacement measured by the transducer, and $\Delta h_{t}^{q}$ is the thermal expansion displacement of the fused quartz glass, which is calculated as:

(3) $$\Delta h_{t}^{q}=\alpha_{q}\;\Delta T\;h_{o}^{q}$$

where ΔT is the temperature difference referred to the room temperature, and $h_{o}^{q}=50\;\mathrm{mm}$ is the initial height of the fused quartz glass.

In this way, the total strain of the sample in the axial direction, can be obtained by:

(4) $$\varepsilon_{h}^{\mathrm{tc}}=\Delta h_{t}^{\mathrm{tot}}/h_{o}^{\mathrm{tot}}$$

where $h_{o}^{\mathrm{tot}}$ is the initial height of the sample.

### 2.4. Creep Test

**Figure 6:**
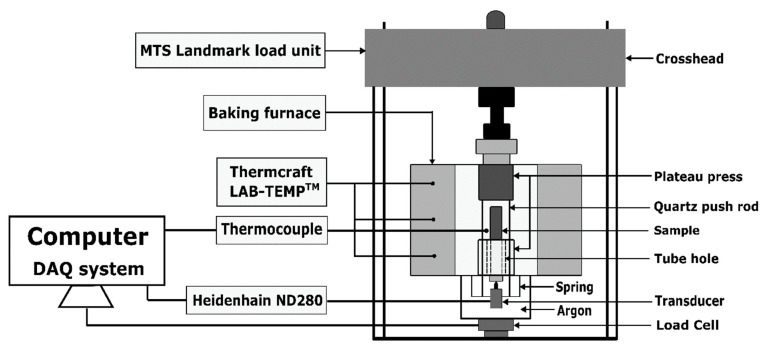
Schematic representation of a pneumatic system for the creep test.

The creep test was performed to study the creep behaviour of the anode at each baking temperature of interest. The test used an in-house pneumatic system as shown in Figure 6, which was established in the Aluminium Research Centre—REGAL at Laval University. The pneumatic system consists of a computer-based DAQ system (DAQ NI USB-6211), an MTS Landmark load unit and a baking furnace connected to a heating device Thermcraft (LAB-TEMP^TM^) (Winston Salem, NC, USA) that can reach a maximum temperature of 1200 °C. The load unit was equipped with two plateau presses. The lower plateau press was fixed to hold the sample while the upper one was lifted down by the crosshead to impose a compressive loading pressure on the sample. The load cell, fixed at the bottom and having a capacity of 5 kN, controlled the loading level. The thermocouple, placed near the sample, measured the sample temperature. Two quartz push-rods were used to transmit the sample displacement to the transducer which was connected to a Heidenhain (ND280) (Traunreut, Bayern, Germany).

During the preheating phase of the anode sample, a preload of 25 N was imposed on the sample. The preload corresponds to the minimum loading capacity of the load unit. Until the sample temperature reached and stabilized at a target temperature, a loading pressure was imposed and kept unchanged for 24 h. To avoid the sample oxidation at high temperatures, argon was injected into the test chamber through the tube holes at a flow rate of 2 L/min.

## 3. Mathematical Models

### 3.1. Baking Index and Shrinking Index

Let us first consider the mass loss fraction of the anode sample written as [34]:

(5) $$x=1-\frac{m^{T}}{m_{o}^{T}}=1-r_{m}$$

where $m^{T}$ is the sample mass at the present temperature; $m_{o}^{T}$ is the initial sample mass and $r_{m}$ is the ratio of sample mass.

In [34], a baking index X was defined as:

(6) $$X=\frac{m_{\mathrm{vf}}}{m_{\mathrm{vf},\infty }}$$

where $m_{\mathrm{vf}}=m_{o}^{T}-m^{T}$ is the mass of released volatile obtained by TGA analysis and “∞” in the subscript denotes the variable value at the end of the devolatilization process. The baking index X characterizes the mass fraction of volatile released in the open pores of the anode.

Using the Equation (5), the baking index can be expressed by the mass loss fraction of the anode sample as:

(7) $$X=\frac{m_{o}^{T}}{m_{\mathrm{vf},\infty }}x$$

The kinetics of the pitch pyrolysis process can be represented by a first-order power equation with respect to the baking index as follows [10]:

(8) $$\frac{dX}{dT}=\frac{k_{o}}{a_{h}}\exp\left(-\frac{E_{a}}{RT}\right)\left(1-X\right)$$

where T is the temperature; $k_{o}$ is the pre-exponential factor; $a_{h}$ is the heating rate; $E_{a}$ is the activation energy and $R=8.314\;J\cdot {\mathrm{mol}}^{-1}\cdot K^{-1}$ is the universal gas constant.

Taking the natural logarithm on both sides of the Equation (8), one can have:

(9) $$\mathrm{In}\left(\frac{dX}{dT}\frac{1}{1-X}\right)=\left(-\frac{E_{a}}{R}\right)\frac{1}{T}+\mathrm{In}\left(\frac{k_{o}}{a_{h}}\right)$$

when the left-hand side of the Equation (9) is expressed as a linear function of (1/T), the activation energy $E_{a}$ and the pre-exponential factor $k_{o}$ can be determined, respectively, by the slope and the intercept.

Using parameters $E_{a}$ and $k_{o}$, the baking index is modeled through an approximate solution of the Equation (8) as [12]:

(10) $$X=1-\exp\left[-\frac{k_{o}RT^{2}}{a_{h}E_{a}}\exp\left(-\frac{E_{a}}{RT}\right)\left(1-\frac{2RT}{E_{a}}\right)\right]$$

However, the definition of the baking index (Equation (6)) does not take into account the entrapped volatile in closed pores of the anode. Accordingly, this baking index does not reflect the real baking level of the anode [27,34]. For this reason, a new variable called the “shrinking index”, was introduced in [27,34]. It was defined as:

(11) $$\chi =\frac{m_{\mathrm{vt}}}{m_{\mathrm{vt},\infty }}$$

where

$$m_{\mathrm{vt}}$$

represents the mass of volatile released in the pore space of the anode including the open pores and the closed pores related to the pitch binder entrapping the volatile.

It is linked with the baking index as follows [27]:

(12) $$\chi =\frac{X}{\zeta }$$

In the Equation (12), ζ is the correlation factor related to the closed and open porosities as follows [27]:

(13) $$\{\begin{array}{l} \zeta =\frac{1+S_{r,\infty }}{1+S_{r}}\\ S_{r}=\tau \frac{\phi_{c}}{\phi_{f}} \end{array}$$

where $S_{r}$ is the ratio of saturation degree; $S_{r,\infty }$ is the ratio of saturation degree at the end of the devolatilization process; τ is the ratio of the volume fraction of the volatile over the corresponding porosities (τ=1 when the volatile fully saturates the open pores and the closed pores related to the pitch binder); $\phi_{c}$ is the closed porosities related to the pitch binder and $\phi_{f}$ is the open porosity. In this work, the open porosity was calculated from the permeability of the anode at high temperatures, which was assumed equal to the air permeability of the anode [34].

### 3.2. Strain Decomposition

During the baking process, the carbon anode is subjected to the thermal expansion, the chemical pyrolysis of the pitch and external loadings. Thus, strains will be induced by these factors. In the case of infinitesimal transformation, the total strain can be additively expressed as:

(14) $$\varepsilon =\varepsilon^{\mathrm{tc}}+\varepsilon^{m}$$

with

(15) $$\{\begin{array}{l} \varepsilon^{\mathrm{tc}}=\varepsilon^{\mathrm{th}}+\varepsilon^{\mathrm{ch}}\\ \varepsilon^{m}=\varepsilon^{e}+\varepsilon^{a}+\varepsilon^{v} \end{array}$$

where $\varepsilon^{\mathrm{th}}$ is the thermal strain; $\varepsilon^{\mathrm{ch}}$ is the chemical strain due to the pitch pyrolysis; $\varepsilon^{e}$ is the elastic strain that characterizes the elastic response; $\varepsilon^{a}$ and $\varepsilon^{v}$ are, respectively, the viscoelastic and viscoplastic strains during the creep behaviour.

#### 3.2.1. Thermal and Chemical Strains

During the dilatometry test, the sample is heated up to a target temperature. Afterwards, the sample is cooled down to estimate the thermal strain, which in turn makes it possible to characterize the thermal expansion coefficient. Since the sample is free of loading, the total strain in the axial direction (Equation (14)) is reduced to the sum of thermal and chemical strains as follows:

(16) $$\varepsilon_{h}=\varepsilon_{h}^{\mathrm{tc}}=\varepsilon_{h}^{\mathrm{th}}+\varepsilon_{h}^{\mathrm{ch}}$$

where $\varepsilon_{h}^{\mathrm{th}}$ and $\varepsilon_{h}^{\mathrm{ch}}$ are axial strains, respectively, due to the thermal expansion and the pitch pyrolysis.

When the anode is cooled down to the room temperature, the total strain in the axial direction (Equation (16)) further reduces to the thermal strain in the axial direction, i.e., $\varepsilon_{h}=\varepsilon_{h}^{\mathrm{th}}$. Therefore, the thermal expansion coefficient at a baking temperature can be estimated using the slope of the thermal strain during the beginning of the cooling phase, as presented below [35]:

(17) $$\alpha_{T}\left(T\right)=\frac{d\varepsilon_{h}^{\mathrm{th}}}{dT}$$

To evaluate the thermal strain $\varepsilon_{h}^{\mathrm{th}}$ at a considered baking temperature $T_{i}$, let us integrate the Equation (17) as follows:

(18) $$\int_{T_{i-1}}^{T_{i}}\alpha_{T}\left(T\right)dT=\int_{T_{i-1}}^{T_{i}}\frac{d\varepsilon_{h}^{\mathrm{th}}}{dT}dT$$

where $T_{i-1}$ the previous considered baking temperature.

The Equation (18) can be approximated by:

(19) $$\varepsilon_{h}^{\mathrm{th}}\left(T_{i}\right)=\varepsilon_{h}^{\mathrm{th}}\left(T_{i-1}\right)+\int_{T_{i-1}}^{T_{i}}\alpha_{T}\left(T\right)dT$$

with

(20) $$\int_{T_{i-1}}^{T_{i}}\alpha_{T}\left(T\right)dT\approx \frac{1}{2}\left[\alpha_{T}\left(T_{i}\right)+\alpha_{T}\left(T_{i-1}\right)\right]\left(T_{i}-T_{i-1}\right)$$

Once the thermal expansion coefficient $\alpha_{T}$ at each baking temperature $T_{i}$ is known, the thermal strain $\varepsilon_{h}^{\mathrm{th}}$ can be obtained by means of Equations (19) and (20). Moreover, the chemical strain $\varepsilon_{h}^{\mathrm{ch}}$ can be further calculated based on the difference between the total strain $\varepsilon_{h}^{\mathrm{tc}}$ and the thermal strain $\varepsilon_{h}^{\mathrm{th}}$.

#### 3.2.2. Mechanical Strains

During the creep test, the impact from the gas pressure in open pores of the anode does not take place since the volatile content in open pores of anode samples have been eliminated by baking the sample to each target temperature before proceeding to the creep test. Furthermore, since the anode undergoes a thermal expansion during the preheating phase, mechanical strains will be extracted from the total strain once the sample temperature stabilizes at a baking temperature. At this moment, the total strain in the Equation (14) is reduced to the sum of mechanical strains:

(21) $$\varepsilon =\varepsilon^{m}=\varepsilon^{e}+\varepsilon^{a}+\varepsilon^{v}$$

**Figure 7:**
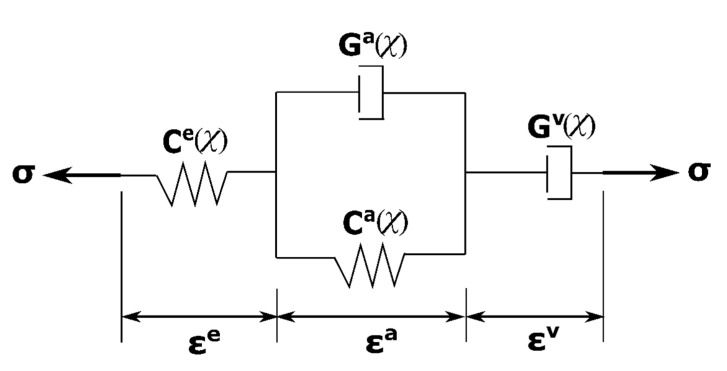
Burger’s rheological model of the anode.

In this work, the Burger’s rheological model is used to represent the viscous behaviour of the anode, as shown in Figure 7. Since parameters involved in the model will be changed by the chemical reaction of pitch pyrolysis, fourth-order tensors involved in the model are considered as a function of the shrinking index [27].

The Burger’s rheological model combines two basic rheological elements: (i) the Kelvin–Voigt element, which describes the viscoelastic behaviour of the material and (ii) the Maxwell element, which describes the elastic and the viscoplastic behaviours of the material. In Figure 7, $\varepsilon^{e}$ characterizes the instantaneous elastic response corresponding to the spring of the Maxwell element; $\varepsilon^{a}$ characterizes the viscoelastic delayed response corresponding to the Kelvin–Voigt element and $\varepsilon^{v}$ characterizes the permanent deformation corresponding to the Maxwell dashpot. According to Figure 7, the formulation of the mechanical behaviour is derived:

(22) $$\{\begin{array}{l} \sigma =C^{e}\left(\chi \right):\varepsilon^{e}\\ \sigma =G^{a}\left(\chi \right):{\dot{\varepsilon }}^{a}+C^{a}\left(\chi \right):\varepsilon^{a}\\ \sigma =G^{v}\left(\chi \right):{\dot{\varepsilon }}^{v} \end{array}$$

where $C^{e}\left(\chi \right)$, $G^{a}\left(\chi \right)$, $C^{a}\left(\chi \right)$ and $G^{v}\left(\chi \right)$ are the fourth-order tensors characterizing, respectively, the elastic behaviour, the viscous effect of the viscoelastic behaviour, the elastic effect of the viscoelastic behaviour and the viscoplastic behaviour at a baking level. Since the material is assumed isotropic, these tensors have the same topology as the elastic tensor such that they are defined as [25]:

(23) $$\{\begin{array}{l} C^{e}\left(\chi \right)=\frac{E_{\chi }^{e}\nu_{\chi }^{e}}{\left(1+\nu_{\chi }^{e}\right)\left(1-2\nu_{\chi }^{e}\right)}I⊗I+\frac{E_{\chi }^{e}}{1+\nu_{\chi }^{e}}\tilde{I}\\ C^{a}\left(\chi \right)=\frac{E_{\chi }^{\mathrm{ae}}\nu_{\chi }^{\mathrm{ae}}}{\left(1+\nu_{\chi }^{\mathrm{ae}}\right)\left(1-2\nu_{\chi }^{\mathrm{ae}}\right)}I⊗I+\frac{E_{\chi }^{\mathrm{ae}}}{1+\nu_{\chi }^{\mathrm{ae}}}\tilde{I}\\ G^{a}\left(\chi \right)=\frac{E_{\chi }^{\mathrm{av}}\nu_{\chi }^{\mathrm{av}}}{\left(1+\nu_{\chi }^{\mathrm{av}}\right)\left(1-2\nu_{\chi }^{\mathrm{av}}\right)}I⊗I+\frac{E_{\chi }^{\mathrm{av}}}{1+\nu_{\chi }^{\mathrm{av}}}\tilde{I}\\ G^{v}\left(\chi \right)=\frac{E_{\chi }^{v}\nu_{\chi }^{v}}{\left(1+\nu_{\chi }^{v}\right)\left(1-2\nu_{\chi }^{v}\right)}I⊗I+\frac{E_{\chi }^{v}}{1+\nu_{\chi }^{v}}\tilde{I} \end{array}$$

where $\left(E_{\chi }^{e},\nu_{\chi }^{e}\right)$ are the Young Modulus and the Poisson’s ratio that describe the elastic behaviour; $\left(E_{\chi }^{\mathrm{ae}},v_{\chi }^{\mathrm{ae}}\right)$ and $\left(E_{\chi }^{\mathrm{av}},\nu_{\chi }^{\mathrm{av}}\right)$ are two pairs of moduli characterizing the viscoelastic behaviour; $\left(E_{\chi }^{v},\nu_{\chi }^{v}\right)$ is a pair of viscous moduli characterizing the viscoplastic behaviour; the subscript “χ” denotes a parameter value as a function of the shrinking index χ; I (${\left(I\right)}_{\mathrm{ij}}=\delta_{\mathrm{ij}}$) and $\tilde{I}$ (${\left(\tilde{I}\right)}_{\mathrm{ijkl}}=\delta_{\mathrm{ik}}\delta_{\mathrm{jl}}$) are, respectively, the second-order and fourth-order identity tensors.

To identify these parameters, the creep test is performed on cylindrical anode samples by a sustained loading stress with free lateral stress. The applied stress tensor is written as:

(24) $$\sigma =\left[\begin{array}{lll} \sigma_{h} & 0 & 0\\ 0 & 0 & 0\\ 0 & 0 & 0 \end{array}\right]$$

where $\sigma_{h}$ is the applied constant axial stress.

The shear strain components equal zero except for the axial and radial strains in the Equation (22). Combining Equations (22)–(24), elastic strains in the axial direction $\varepsilon_{h}^{e}$ and in the radial direction $\varepsilon_{r}^{e}$ are expressed as:

(25) $$\{\begin{array}{l} \varepsilon_{h}^{e}=\frac{\sigma_{h}}{E_{\chi }^{e}}\\ \varepsilon_{r}^{e}=-\frac{\nu_{\chi }^{e}\sigma_{h}}{E_{\chi }^{e}} \end{array}$$

Developing the stress-strain relationship corresponding to the Kelvin–Voigt model (Equation (22)), and taking into account definitions of fourth-order tensor $G^{a}\left(\chi \right)$ and $C^{a}\left(\chi \right)$, one can show that viscoelastic strains in axial and radial directions $\varepsilon_{h}^{a}$ and $\varepsilon_{r}^{a}$ are expressed as [25]:

(26) $$\{\begin{array}{l} \varepsilon_{h}^{a}=\sigma_{h}\left(\frac{1}{9}{\left(K_{\chi }^{a}\right)}^{-1}\frac{1-e^{-\lambda_{1}t}}{\lambda_{1}}+\frac{1}{3}{\left(G_{\chi }^{a}\right)}^{-1}\frac{1-e^{-\lambda_{2}t}}{\lambda_{2}}\right)\\ \varepsilon_{r}^{a}=\sigma_{h}\left(\frac{1}{9}{\left(K_{\chi }^{a}\right)}^{-1}\frac{1-e^{-\lambda_{1}t}}{\lambda_{1}}-\frac{1}{6}{\left(G_{\chi }^{a}\right)}^{-1}\frac{1-e^{-\lambda_{2}t}}{\lambda_{2}}\right) \end{array}$$

with

(27) $$\{\begin{array}{l} {\left(K_{\chi }^{a}\right)}^{-1}=\frac{3\left(1-2\nu_{\chi }^{\mathrm{av}}\right)}{E_{\chi }^{\mathrm{av}}}\\ {\left(G_{\chi }^{a}\right)}^{-1}=\frac{2\left(1+\nu_{\chi }^{\mathrm{av}}\right)}{E_{\chi }^{\mathrm{av}}}\\ \lambda_{1}=\frac{E_{\chi }^{\mathrm{ae}}\left(1-2\nu_{\chi }^{\mathrm{av}}\right)}{E_{\chi }^{\mathrm{av}}\left(1-2\nu_{\chi }^{\mathrm{ae}}\right)}\\ \lambda_{2}=\frac{E_{\chi }^{\mathrm{ae}}\left(1+\nu_{\chi }^{\mathrm{av}}\right)}{E_{\chi }^{\mathrm{av}}\left(1+\nu_{\chi }^{\mathrm{ae}}\right)} \end{array}$$

where ${\left(K_{\chi }^{a}\right)}^{-1}$ and ${\left(G_{\chi }^{a}\right)}^{-1}$ are constants related to hydrostatic and deviatoric viscoelastic behaviour [25]; and $\lambda_{1}$ and $\lambda_{2}$ are coefficients related to the relaxation time corresponding to the viscoelastic behaviour of the material.

Integrating both sides of the third line in the Equation (22) and using the Equation (23), viscoplastic strains in axial and radial directions can be calculated as follows:

(28) $$\{\begin{array}{l} \varepsilon_{h}^{v}=\frac{1-{\left(\nu_{\chi }^{v}\right)}^{2}}{E_{\chi }^{v}}\sigma_{h}t\\ \varepsilon_{r}^{v}=-\frac{\nu_{\chi }^{v}\left(1+\nu_{\chi }^{v}\right)}{E_{\chi }^{v}}\sigma_{h}t \end{array}$$

From Equations (26) and (28), the creep strains of the anode sample in axial and radial directions, corresponding to the sum of viscoelastic and viscoplastic strains, can be expressed as:

(29) $$\{\begin{array}{l} \varepsilon_{h}^{c}=\sigma_{h}\left[\frac{1}{9}{\left(K_{\chi }^{a}\right)}^{-1}\frac{1-e^{-\lambda_{1}t}}{\lambda_{1}}+\frac{1}{3}{\left(G_{\chi }^{a}\right)}^{-1}\frac{1-e^{-\lambda_{2}t}}{\lambda_{2}}+\frac{1-{\left(\nu_{\chi }^{v}\right)}^{2}}{E_{\chi }^{v}}t\right]\\ \varepsilon_{r}^{c}=\sigma_{h}\left[\frac{1}{9}{\left(K_{\chi }^{a}\right)}^{-1}\frac{1-e^{-\lambda_{1}t}}{\lambda_{1}}-\frac{1}{6}{\left(G_{\chi }^{a}\right)}^{-1}\frac{1-e^{-\lambda_{2}t}}{\lambda_{2}}-\frac{\nu_{\chi }^{v}\left(1+\nu_{\chi }^{v}\right)}{E_{\chi }^{v}}t\right] \end{array}$$

## 4. Results and Discussion

### 4.1. Thermogravimetric Analysis and Shrinking Index

**Figure 8:**
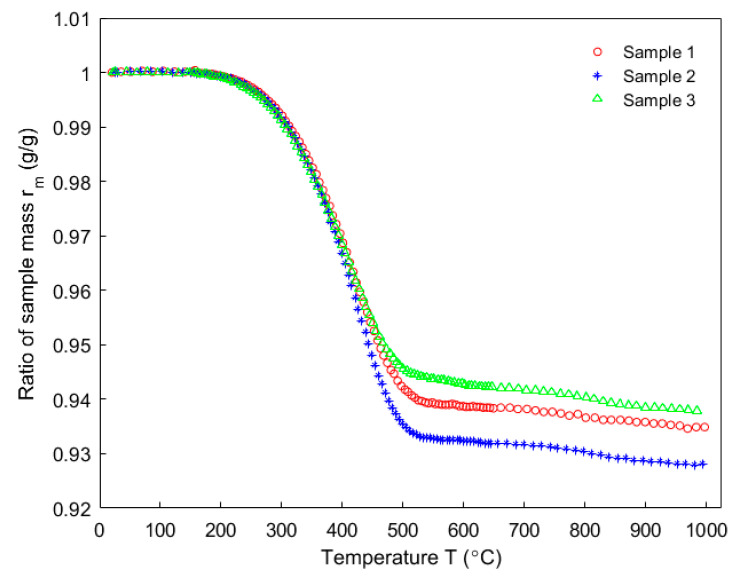
Evolution of sample mass as a function of the temperature.

Figure 8 shows the evolution of the ratio of sample mass, obtained by using three different samples for the thermogravimetric analysis from room temperature to 1000 °C. As the figure shows, the evolutions of all are almost similar although each sample has a different initial mass.

**Figure 9:**
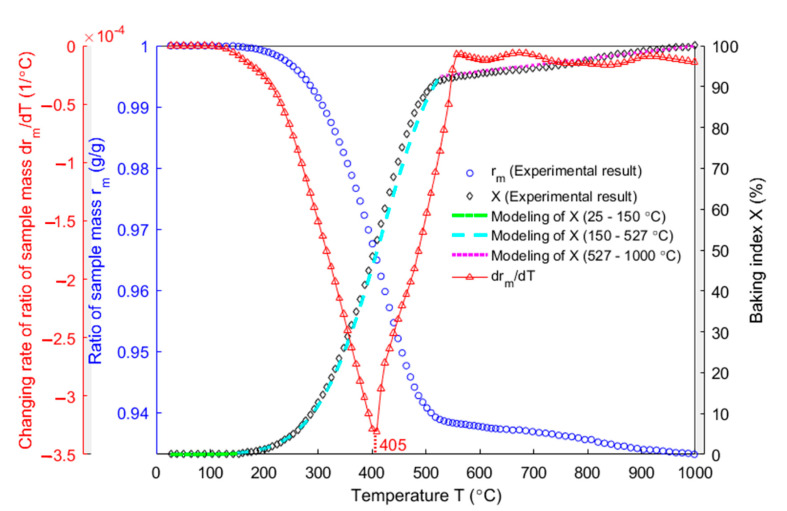
Evolution of the ratio of sample mass, the changing rate of the ratio of sample mass, the baking index and the modeling of baking index as a function of temperature.

Figure 9 shows the evolution of the ratio of sample mass (blue circle line), the changing rate of the ratio of sample mass (red upper-pointing triangular line), the baking index (black diamond line) and the modeling of baking index (green, cyan and magenta lines). The sample mass used in Figure 9 is the average mass of three samples. To reduce the oscillation in the ratio of sample mass, the experimental data is smoothed by using the built-in function ‘rlowess’ in MATLAB R2016a. The changing rate of the ratio of sample mass is then obtained as the first derivative of the smoothed curve. From the ratio of sample mass, the anode starts losing weight from around 150 °C and the mass decreases by raising the baking temperature. From the baking index, 91.4% of released volatile is devolatilized from 150 to 527 °C with 6.11% of sample mass having been lost. The minimum value of the changing rate of the ratio of sample mass appears at 405 °C. The anode slows down losing the mass during the subsequent baking. At the end of the baking process, the anode will have lost 10.1 g of mass in form of the volatile which occupies 6.7% of the total sample mass.

From 527 to 627 °C, only 0.15% of mass will be lost, which is indicative of the formation of semi-coke [22]. Upon further baking from 627 to 1000 °C, only 0.44% of total mass will be lost, which indicates that the solid green coke is produced from the semi-coke obtained at the temperatures below 627 °C [22].

**Figure 10:**
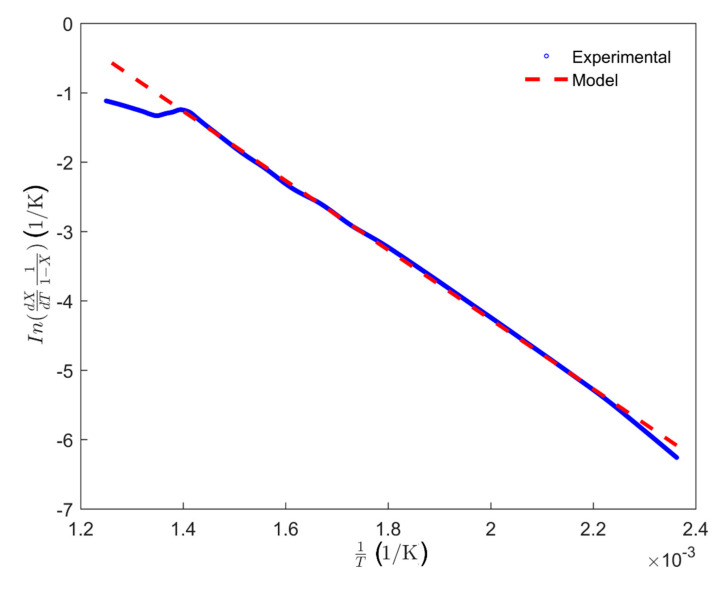
Modeling of the evolution of the quantity $\ln\left(\frac{dX}{dT}\frac{1}{1-X}\right)$ as a function of $\frac{1}{T}$ in determining activation energy and pre-exponential factor.

Since no volatile evolves from the anode between 25 and 150 °C, the modeling of the baking index within this temperature range (green dash-dot line) equals zero. Between 150 and 527 °C (cyan dashed line), the baking index was modeled by the Equation (10). To this end, the estimated baking index within this temperature range (Figure 9) was used to plot the evolution of the quantity $\ln\left(\frac{dX}{dT}\frac{1}{1-X}\right)$ as a function of $\frac{1}{T}$ (Equation (9)) with the temperature unit in Kelvin, which was presented as the blue dotted line in Figure 10. This evolution was modelled by a linear function, shown as the red dashed line in Figure 10. Afterwards, the activation energy $E_{a}=41.41\;\mathrm{kJ}\cdot {\mathrm{mol}}^{-1}$ and the pre-exponential factor $k_{o}=299.3\;h^{-1}$ were, respectively, calculated from the slope and the intercept of the linear curve (red dashed line) according to the Equation (9).

From 527 to 1000 °C (magenta dotted line), little volatile escapes from the anode (8.6% of released volatile that has escaped from the anode) and the behaviour of the baking index is almost linear. As a result, the modeling of the baking index is assumed linear within this temperature range, as indicated below:

(30) X=aT+b (527 °C−1000 °C)

where $a=1.56\times 10^{-4}\;{}^{\circ}C^{-1}$ and b=84.3 % are, respectively, the slope and the intercept of the model of the baking index within the temperature of 527 to 1000 °C.

**Figure 11:**
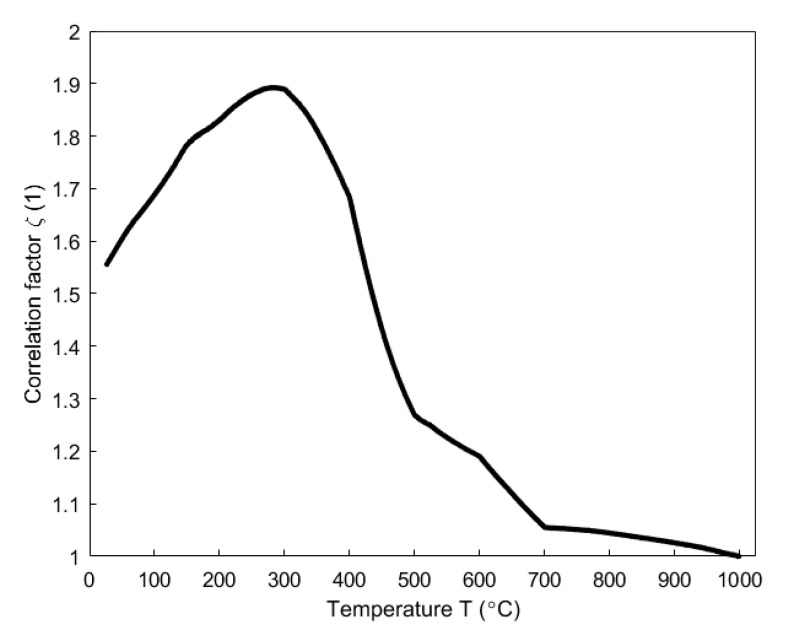
Evolution of the correlation factor as a function of temperature [34].

**Figure 12:**
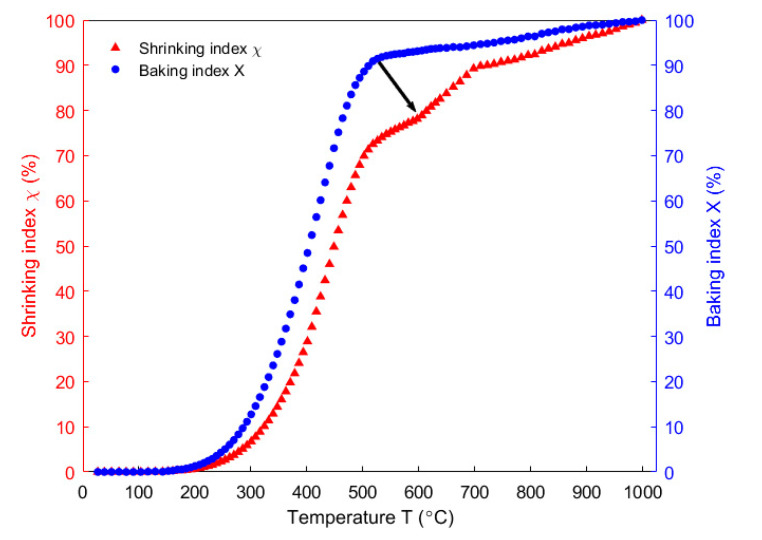
Evolution of the shrinking index and the baking index as a function of temperature.

In [34], the correlation factor ζ in the Equation (12) was obtained from the open and closed porosities related to the pitch binder, as shown in Figure 11. In this case, the permeability at high temperatures was assumed equal to the air permeability (the permeability correlator was assumed equal to one). Then, according to the Equation (12), the shrinking index was calculated using the baking index (Figure 9), as shown in Figure 12. The figure shows that, the shrinking index (red upper-pointing triangles) deviates from the baking index (blue dots). This deviation especially takes place within the temperature range of 300–500 °C where phenomena such as the thermal expansion, chemical shrinkage, maximum releasing rate of the volatile and maximum pressure in closed pores related to the pitch binder occur [34]. Such a deviation is due to the closed porosities associated with the pitch binder that were introduced in the definition of the shrinking index. Accordingly, the shrinking index seems to better reflect the baking level than the baking index.

### 4.2. Dilatometry

**Figure 13:**
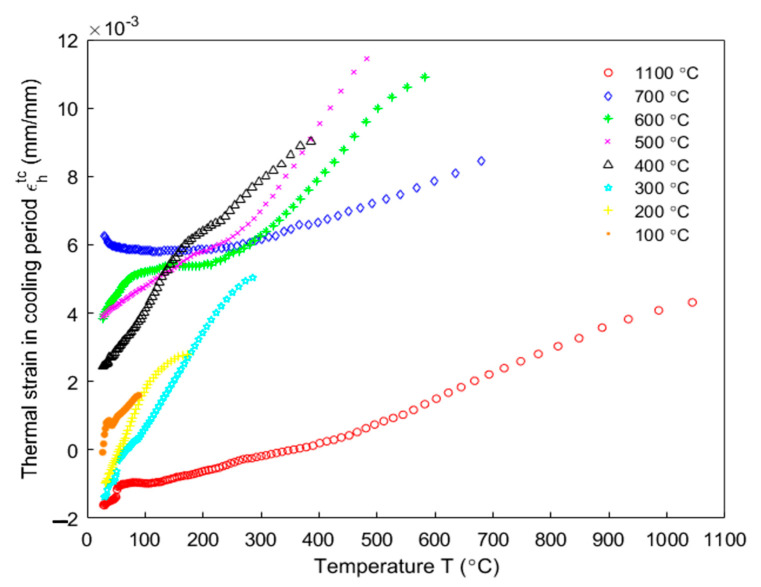
Thermal strain of the sample in the axial direction during the cooling down to the room temperature for different target temperatures ranging from 100 to 1100 °C.

The dilatometry was used to measure anode displacements in the axial direction for different target temperatures. For each target temperature, two tests were performed and each test used one green anode sample. The mean value of obtained results was calculated for each time point and it was used to represent the evolution of the total strain of the anode in the axial direction. Figure 13 shows the axial strain during the cooling period for target temperatures ranging from 100 to 1100 °C. The sample does not undergo any chemical reaction during this period, hence only the thermal strain takes place. Furthermore, one can notice that the thermal strain is a nonlinear function of the temperature. The thermal expansion coefficient $\alpha_{T}$ was obtained by calculating the slope of a line segment from the beginning of the cooling period for each target temperature. The thermal strain was obtained using Equations (19) and (20).

**Figure 14:**
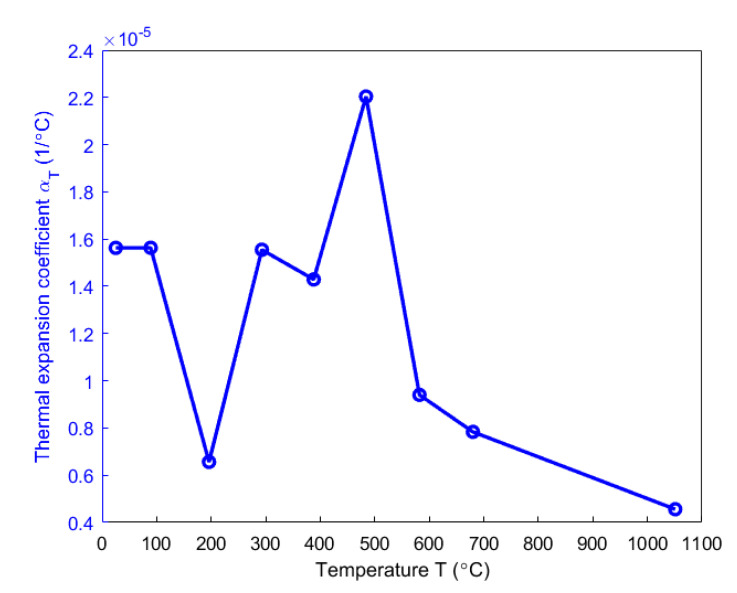
Evolution of thermal expansion coefficient with respect to temperature.

Figure 14 shows the evolution of the thermal expansion coefficient. This coefficient reaches the maximum value of 2.204 × 10^−5^ °C^−1^ at the target temperature of 500 °C. For target temperatures ranging from 25 to 100 °C, the thermal expansion coefficient is assumed to remain the same since no chemical reaction takes place within this temperature range. The decrease in the thermal expansion coefficient for target temperatures ranging from 100 to 200 °C and its increase from 200 to 500 °C might be due to the pitch softening and the pitch carbonization, respectively. For target temperatures ranging from 500 to 1100 °C, the decreasing trend corroborates the observation in [15] of a carbon mixture of pitch and coke particles. Microscopically, this decrease is due to the transmitting expansion power of the micro-crystallites in the c-direction of a ring structure and the restraining influence of the ring structure of interconnected micro-crystallites from their expansion [15]. At the end of the baking, the prebaked anode will have a thermal expansion coefficient of 4.56 × 10^−6^ °C^−1^ which is nearly in the magnitude of values ranging between 3.75 × 10^−6^ and 4.5 × 10^−6^ °C^−1^ given by [36].

**Figure 15:**
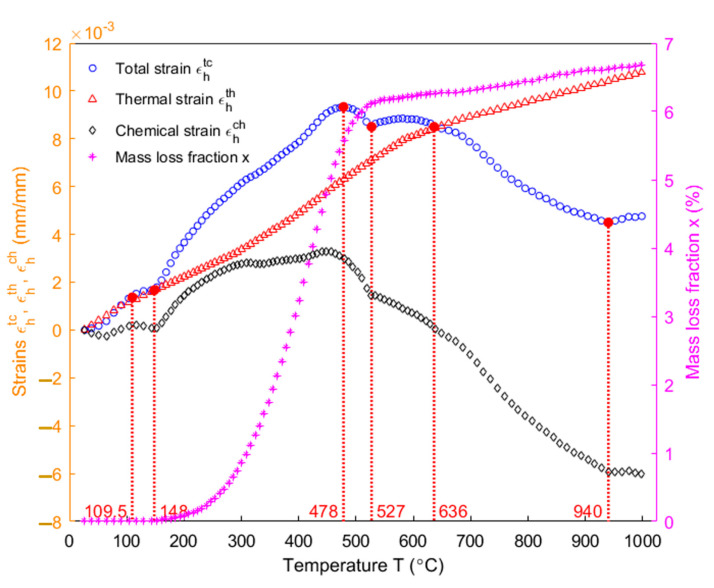
Evolution of the total strain, the thermal strain, the chemical strain of the sample in the axial direction and the mass loss fraction as a function of temperature.

In Figure 15, the total strain and the thermal strain (Equations (19) and (20)) of the sample in the axial direction are, respectively, represented by blue dots and red upper-pointing triangles. The chemical strain (black diamonds) is obtained as the difference between the two. The mass loss fraction of the anode (magenta asterisks) is obtained using the ratio of sample mass (Figure 9) according to the Equation (5). Below 148 °C, the total strain almost coincides with the thermal strain and the anode does not lose any mass according to the mass loss fraction of the anode. Thus, no chemical strain takes place, as shown in Figure 15. From the room temperature to 109.5 °C (softening point of the pitch), the sample expands due to the thermal expansion of the pitch and coke aggregates. From 109.5 to 148 °C, the expansion of the sample is slowed down by the pitch softening. From 148 to 478 °C, the pitch pyrolyzes and the entrapped volatile in closed pores significantly contributes to the sharp increase in the total strain in this temperature range [34]. At 478 °C, the total strain reaches its maximum value which almost coincides with the maximum chemical strain at 450 °C. Afterwards, the total strain decreases with decrease in the chemical strain, which indicates that the chemical shrinkage has begun to take effect. However, the hump of the total strain taking place between 527 and 636 °C (Figure 15) is caused by the internal gas pressure due to the devolatilization process and the mass loss from the anode, even though the chemical shrinkage takes effect [30,34]. This demonstrates that the decrease in the chemical strain slows down in this temperature range. Between 148 and 636 °C, one can notice that the chemical strain is positive and that the majority of gaseous volatile is generated. This implies that, between these two temperatures, the chemical strain mainly affects the total strain through the internal gas pressure. Above 636 °C, the chemical strain becomes negative, indicating that the impact of the internal gas pressure is negligible and the chemical shrinkage of the pitch binder due to the carbonization of the pitch dominates in decreasing the total strain. As a result, the thermal strain exceeds the total strain. Therefore, one can conclude that the chemical strain is a superimposition of the strain due to the increase in the pore pressure caused by the entrapped volatile and the strain due to the chemical shrinkage caused by the pitch carbonization. Above 940 °C, the chemical shrinkage is considerably weakened and the expansion of the material is mainly due to the thermal expansion [34].

### 4.3. Elastic Parameters and Compressive Strength

**Figure 16:**
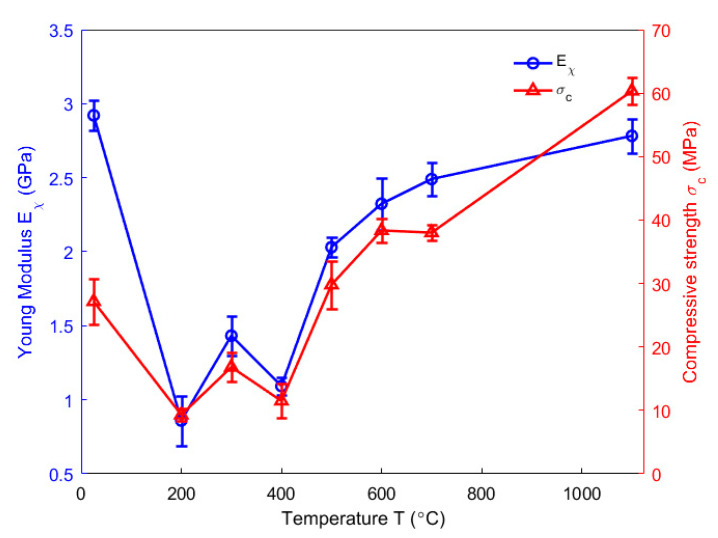
Evolution of Young Modulus and compressive strength as a function of temperature [37].

Mechanical parameters associated with the spring of the Maxwell element in Burger’s rheological model are indicative of elastic deformation that would be instantaneously recovered after eliminating the applied stress. According to the Equation (25), the Poisson’s ratio characterizes the elastic behaviour of the anode in the radial direction. However, it was not characterized in this work due to the difficulties of measuring radial strains of the anode sample at high temperatures. In [37], the compression tests at several high temperatures were accomplished. To eliminate the volatile content in open pores, the samples were baked to each high temperature ranging from 200 to 1100 °C and cooled down. At each high temperature level, the Young Modulus was obtained by applying three loading/unloading cycles according to the ASTM standard C469/469M-14 [38] and the compressive strength was determined by starting a loading until the rupture of the sample [37]. Three tests were repeated at each baking temperature. The mean value of obtained results was used to represent the Young Modulus and the compressive strength of the carbon anode in the axial direction. Figure 16 shows the results of Young Modulus and compressive strength as a function of temperature. As seen in the figure, these two greatly decrease below 200 °C due to the pitch softening while their variations above 200 °C are mainly caused by the pitch pyrolysis process. From 200 to 400 °C, the transformation of the structure and the physical properties of crystalline carbon in the mesophase transition [2] probably changes the values of Young Modulus and compressive strength within this temperature range. Since the anode material used in this work has similar material components with the carbon material characterized in [39], the increase in the compressive strength above 400 °C might be explained by the presence of large thermal stresses after cooling from the baking temperature [39]. Moreover, the difference between the thermal expansion of coke particles and the pitch binder might close up some pores and squeeze the coke particles together more tightly, thus, the Young Modulus increases above 400 °C [39].

### 4.4. Creep Test and Inverse Identification of Parameters

**Figure 17:**
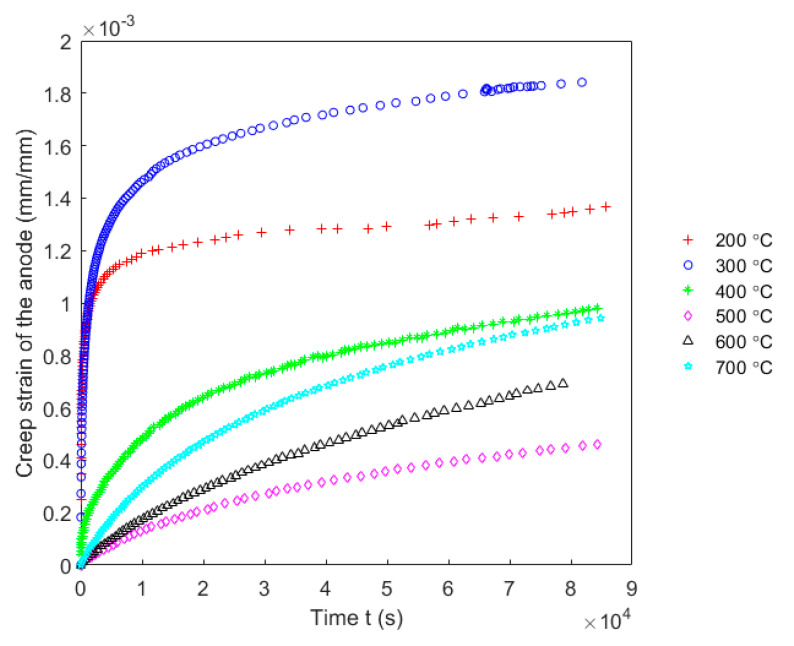
Creep strains of the carbon anode in the axial direction at different baking temperatures ranging from 200 to 700 °C.

Creep tests were carried out to characterize the viscous behaviour of the anode in the axial direction at high temperatures. To this end, creep tests were done under a stress level of $\sigma_{h}=50\;\mathrm{kPa}$. However, due to difficulties of measuring operations at high temperatures, radial strains were not measured. At each baking temperature, two tests were performed and each test used one baked-up sample to that baking temperature. The mean value of obtained results was calculated for each time point and was used to represent the evolution of the creep strain of the carbon anode in the axial direction. Figure 17 shows the evolution of creep strains of the carbon anode in the axial direction as a function of time at different baking temperatures ranging from 200 to 700 °C. As the figure indicates, the creep strain is very sensitive to the baking temperature. The creep strain at the baking temperature 300 °C is the highest while at the baking temperature 500 °C it is the lowest among these temperature levels.

**Figure 18:**
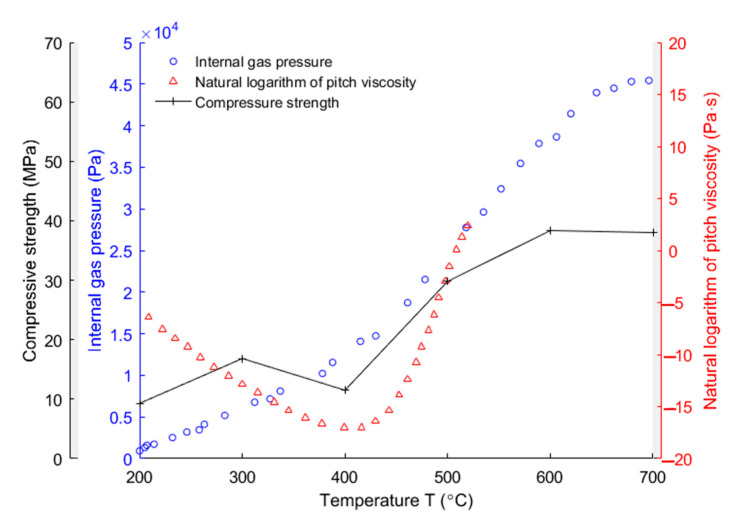
Internal gas pressure [30], natural logarithm of pitch viscosity [2] and compressive strength of the anode [37].

In this work, the creep behaviour of the anode at the baking temperatures ranging from 200 to 700 °C is considered to be impacted by three properties: (i) the internal gas pressure due to the devolatilization process; (ii) the change of pitch viscosity due to the phase transformation at high temperatures and (iii) the compressive strength of the anode. Figure 18 shows the evolutions of these properties as a function of temperature between 200 and 700 °C according to [2,30,37]. We can see in the figure that the internal gas pressure increases from 200 to 700 °C. The natural logarithm of pitch viscosity decreases from −6.4 Pa·s at 208 °C to −17 Pa·s at 400 °C, and afterwards, increases to 2.4 Pa·s at 520 °C. Moreover, it can be noticed that the compressive strength of the anode significantly increases from 400 to 600 °C.

The creep strain at 200 °C is lower than the creep strain at 300 °C. This could be due to the decrease in pitch viscosity between 200 and 300 °C when the internal gas pressure and the compressive strength of the anode, as shown in Figure 18, are still low within this temperature range. From 300 to 400 °C, since the pitch viscosity and the compressive strength of the anode are low and there is less connectivity between open pores and closed bubble pores in the anode within this temperature range, the decrease in the creep strain from 300 to 400 °C is mainly due to the resistance of the increase in the internal gas pressure caused by the volatile entrapment to the external loading [6,34]. From 400 to 500 °C, the pitch viscosity and the compressive strength of the anode sharply increase even though the internal gas pressure still increases within this temperature range, as shown in Figure 18. Thus, the decrease in the creep strain from 400 to 500 °C is mainly due to the increase in the pitch viscosity and the compressive strength of the anode. From 500 to 600 °C, the chemical shrinkage of the pitch binder links the open pores with the bubble closed pores [40], which consequently facilitates the diffusion of the volatiles out of the anode. Thus, the internal gas pressure, as shown in Figure 18, significantly increases due to the volatile diffusion. In this case, the material becomes more inclined to deform under the external loading. Thus, the creep strain at 600 °C is larger than that at 500 °C. The increase in the creep strain from 600 to 700 °C might be due to the further increase in the internal gas pressure caused by the volatile diffusion when the compressive strength of the anode remains almost constant within this temperature range, as can be seen in Figure 18.

To assess the predictive capabilities of Burger’s rheological model, an inverse identification procedure was carried out to determine its material parameters for different baking temperatures. To this end, for each baking temperature $T_{i}$, an objective function was defined as follows:

(31) $$f_{T_{i}}\left(E_{\chi }^{\mathrm{av}},E_{\chi }^{\mathrm{ae}},E_{\chi }^{v},\nu_{\chi }^{\mathrm{av}},\nu_{\chi }^{\mathrm{ae}},\nu_{\chi }^{v}\right)=\sum_{j=1}^{n_{T_{i}}}{\left({\left(\varepsilon_{h}^{c}\right)}_{j}^{\exp}-{\left(\varepsilon_{h}^{c}\right)}_{j}^{\mathrm{model}}\right)}^{2}$$

where $n_{T_{i}}$ is the number of experimental data measured during the creep period; ${\left(\varepsilon_{h}^{c}\right)}_{j}^{\exp}$ is the j-th value of the experimental creep strain in the axial direction and ${\left(\varepsilon_{h}^{c}\right)}_{j}^{\mathrm{model}}$ is the associated model’s creep strain.

The pattern search algorithm built-in MATLAB R2016a was used for minimizing the objective function [41]. The obtained model’s parameters for baking temperatures from 200 to 700 °C are summarized in Table 3. Notice that only the axial creep strains were used to define the objective function (Equation (31)). Therefore, one can expect this inverse identification procedure to be insensitive to the Poisson-like parameters $\nu_{\chi }^{\mathrm{av}}$, $\nu_{\chi }^{\mathrm{ae}}$ and $\nu_{\chi }^{v}$ since they are related to the radial strains which were not measured in this study [24]. Accordingly, obtained values corresponding to these Poisson-like parameters may not reflect their real values. In the Appendix A, a sensitivity analysis was performed to assess the effect of parameters $\nu_{\chi }^{\mathrm{av}}$, $\nu_{\chi }^{\mathrm{ae}}$ and $\nu_{\chi }^{v}$ on the inverse identification procedure. It was shown that by constraining the parameters $\nu_{\chi }^{\mathrm{av}}$, $\nu_{\chi }^{\mathrm{ae}}$ and $\nu_{\chi }^{v}$ to vary within several different intervals belonging to the interval [0, 0.5], minimization of the objective function (Equation (31)) leads to almost identical values for parameters $E_{\chi }^{\mathrm{av}}$, $E_{\chi }^{\mathrm{ae}}$ and $E_{\chi }^{v}$ while those for parameters $\nu_{\chi }^{\mathrm{av}}$, $\nu_{\chi }^{\mathrm{ae}}$ and $\nu_{\chi }^{v}$ are not unique.

**Figure 19:**
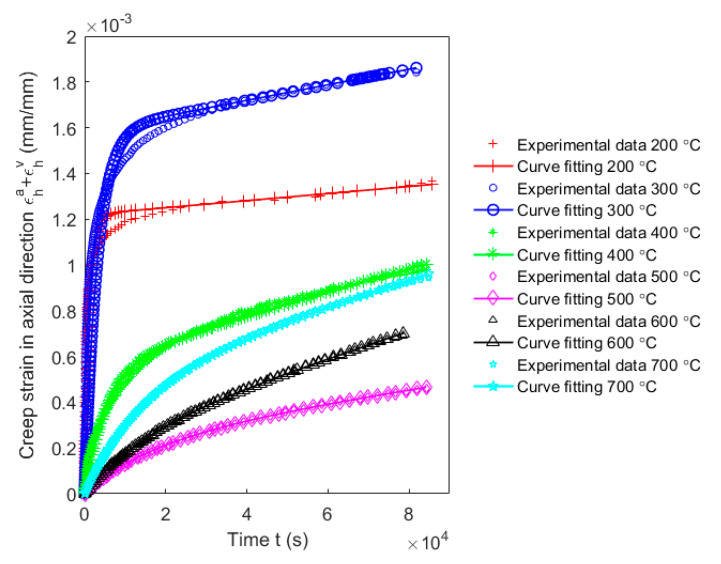
Model prediction of the creep behaviour of carbon anode in the axial direction for different baking temperatures.

The model results of the creep behaviour using these parameters for baking temperatures from 200 to 700 °C are compared with the obtained experimental findings in Figure 19. It is observed in the figure that the model well represents the creep behaviour of the carbon anode in the axial direction for all baking temperatures.

**Figure 20:**
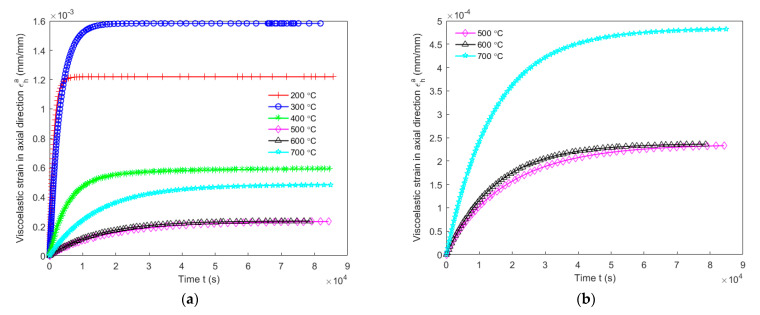
Modeling of viscoelastic strain of the carbon anode in the axial direction: (**a**) baking temperatures 200 °C–700 °C; (**b**) baking temperatures 500 °C, 600 and 700 °C.

**Figure 21:**
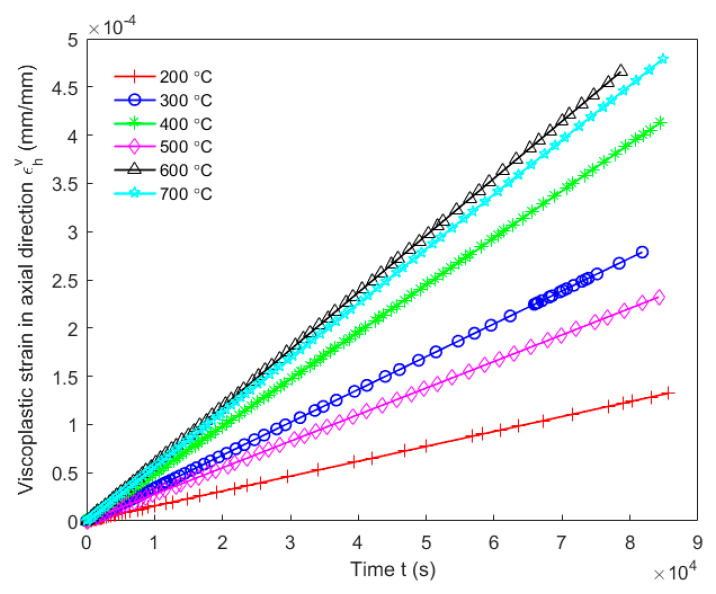
Modeling of viscoplastic strain of the carbon anode in the axial direction for different baking temperatures.

Figure 20 and Figure 21 show, respectively, the modeling of the viscoelastic behaviour and the viscoplastic behaviour of the anode during the creep period according to Equations (26) and (28). It can be observed in Figure 20 that the viscoelastic behaviour of the anode at the baking temperature 200 °C reaches its asymptotical value much faster than at the other temperatures, and that it takes the longest time for the viscoelastic behaviour at the baking temperature 500 °C to reach its asymptotical value compared to the other temperatures. As illustrated in Figure 21, it was found that the magnitude of the viscoplastic behaviour of the anode during the creep period is the highest at the baking temperature 600 °C and is the lowest at the baking temperature 200 °C.

**Figure 22:**
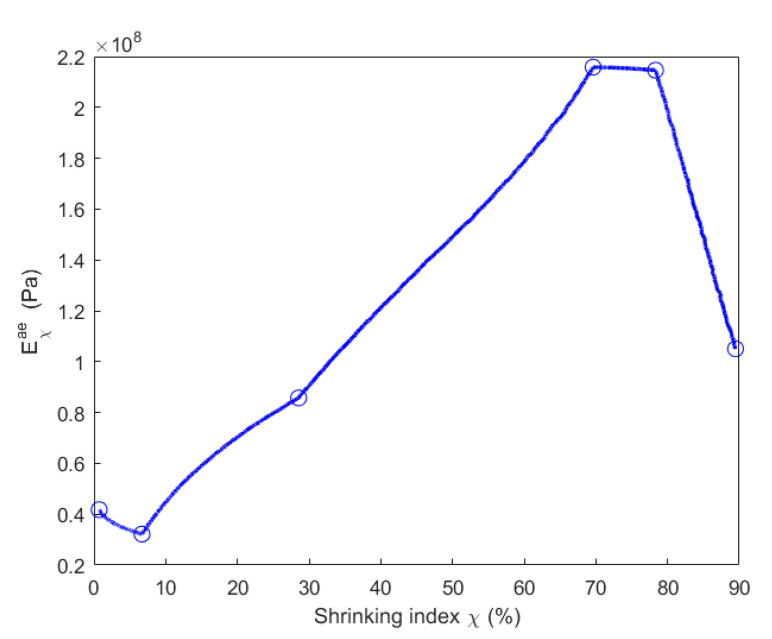
Evolution of the parameter $E_{\chi }^{\mathrm{ae}}$ with respect to the shrinking index.

**Figure 23:**
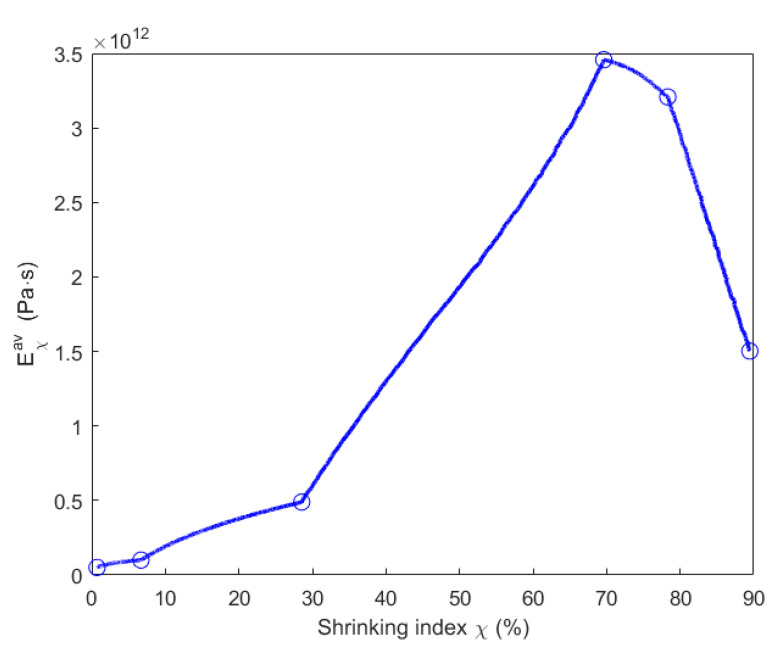
Evolution of the parameter $E_{\chi }^{\mathrm{av}}$ with respect to the shrinking index.

**Figure 24:**
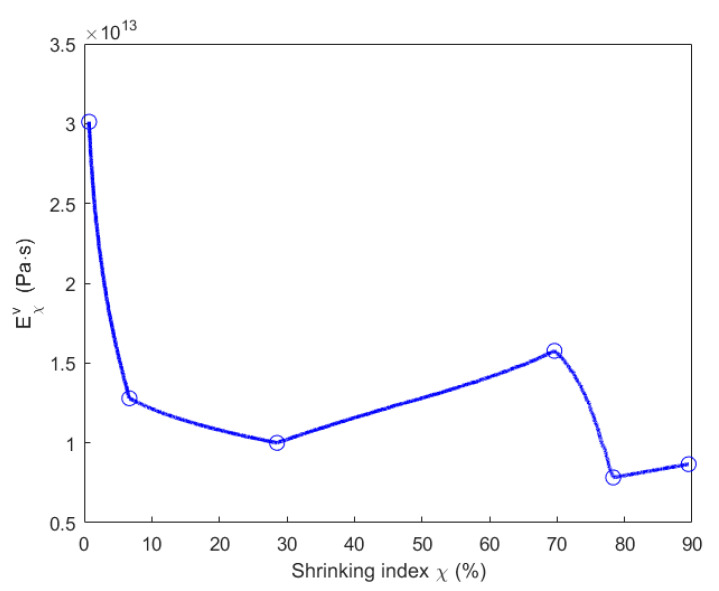
Evolution of the parameter $E_{\chi }^{v}$ with respect to the shrinking index.

Taking into account the chemo-mechanical coupling effect when the material is degraded by the chemical pyrolysis, parameters $E_{\chi }^{\mathrm{ae}}$, $E_{\chi }^{\mathrm{av}}$ and $E_{\chi }^{v}$ presented in Table 3 are expressed as a function of the shrinking index, as shown in Figure 22, Figure 23 and Figure 24.

According to Equations (26) and (27), when t→∞, the asymptotical value of viscoelastic strain in the axial direction $\varepsilon_{h,\infty }^{a}$ is obtained as:

(32) $$\varepsilon_{h,\infty }^{a}=\frac{\sigma_{h}}{E_{\chi }^{\mathrm{ae}}}$$

Considering the Equation (32), one can notice that $E_{\chi }^{\mathrm{ae}}$ controls the magnitude of the asymptotical value of viscoelastic strain in the axial direction under a constant loading $\sigma_{h}$ at different baking temperatures. Furthermore, according to the Equation (27), $E_{\chi }^{\mathrm{ae}}/E_{\chi }^{\mathrm{av}}$ mainly affects the relaxation time through coefficients $\lambda_{1}$ and $\lambda_{2}$.

Figure 20 indicates that $\varepsilon_{h,\infty }^{a}$ is larger at the baking temperature 300 °C than at 200 °C. Thus, $E_{\chi }^{\mathrm{ae}}$ decreases from 0.7% (200 °C) to 6.7% (300 °C) of shrinking index in Figure 22. Moreover, as shown in Figure 20, $\varepsilon_{h}^{a}$ at 200 °C increases faster to the asymptotical value than it does at 300 °C because of the decrease in $\lambda_{1}$ and $\lambda_{2}$ between 200 and 300 °C according to Equations (26) and (27). Thus, $E_{\chi }^{\mathrm{av}}$ increases from 0.7% (200 °C) to 6.7% (300 °C) of shrinking index in Figure 23. Since $\varepsilon_{h,\infty }^{a}$ decreases from 300 to 500 °C in Figure 20, $E_{\chi }^{\mathrm{ae}}$ increases from 6.7% (300 °C) to 70% (500 °C) of shrinking index in Figure 22. Furthermore, asymptotical $\varepsilon_{h}^{a}$ slows down because of the decrease in $\lambda_{1}$ and $\lambda_{2}$ between 300 and 500 °C. Thus, $E_{\chi }^{\mathrm{av}}$ increases from 6.7% (300 °C) to 70% (500 °C) of shrinking index in Figure 23. For baking temperatures 500 and 600 °C, the values of $\varepsilon_{h,\infty }^{a}$ are almost the same in Figure 20. Thus, $E_{\chi }^{\mathrm{ae}}$ at these two temperatures are almost the same, as shown in Figure 22. Moreover, the increasing speed of $\varepsilon_{h}^{a}$ to its asymptotical value is higher at 600 °C than at 500 °C because of the increase in $\lambda_{1}$ and $\lambda_{2}$ from 500 to 600 °C. This leads to a decrease in the value of $E_{\chi }^{\mathrm{av}}$ from 70 % (500 °C) to 78% (600 °C) of shrinking index, as demonstrated in Figure 23. For the baking temperature ranging from 600 to 700 °C, the increase in $\varepsilon_{h,\infty }^{a}$ leads to the decrease in $E_{\chi }^{\mathrm{ae}}$ from 78% (600 °C) to 89% (700 °C) of shrinking index in Figure 22 and the increasing speed of $\varepsilon_{h}^{a}$ to its asymptotical value rises in Figure 20 because of the increase in $\lambda_{1}$ and $\lambda_{2}$ from 600 to 700 °C. This causes further decrease in $E_{\chi }^{\mathrm{av}}$ from 78% (600 °C) to 89% (700 °C) of shrinking index, as shown in Figure 23.

Since $\nu_{\chi }^{v}$ varies within [0, 0.5], the variation of ($1-{\left(\nu_{\chi }^{v}\right)}^{2}$) lies in the range [0.75, 1]. Thus, the increasing speed of the viscoplastic strain mainly depends on $E_{\chi }^{v}$ according to the Equation (28). In Figure 21, for the baking temperature ranging from 200 to 400 °C, the increasing magnitude of the viscoplastic strain indicates a decrease in $E_{\chi }^{v}$ from 0.7% (200 °C) to 29% (400 °C) of shrinking index in Figure 24. For the baking temperature ranging from 400 to 500 °C, the magnitude of viscoplastic strain decreases, hence $E_{\chi }^{v}$ increases from 29% (400 °C) to 70% (500 °C) of shrinking index in Figure 24. For the baking temperature ranging from 500 to 600 °C, the increasing magnitude of viscoplastic strain leads to a decrease in $E_{\chi }^{v}$ from 70% (500 °C) to 78% (600 °C) of shrinking index in Figure 24. For the baking temperature ranging from 600 to 700 °C, the decreasing magnitude of viscoplastic strain increases $E_{\chi }^{v}$ from 78% (600 °C) to 89% (700 °C) of shrinking index in Figure 24.

**Remark:**  *Parameters involved in the mathematical model have been summarized in Appendix B.*

## 5. Conclusions

In this work, thermo-chemo-mechanical properties of the anode mixture at high temperatures were characterized. To this end, experiments consisting of thermogravimetric analysis, dilatometry and creep tests were carried out. A high heating rate for the sample baking was used to highlight the effect of the pitch pyrolysis reaction on the thermo-mechanical behaviour of the anode during the baking process.

The obtained results allow for the characterization of the anode mass, the thermal strain, the chemical strain, and the creep strains of the anode at different high temperature levels. At first, the evolution of the baking index was obtained using the anode mass. Using the baking index, the activation energy and the pre-exponential factor involved in the power-law model between 150 and 527 °C were identified. Additionally, the slope and the intercept of the linear model between 527 and 1000 °C were identified. These parameters provide a way to investigate the thermal-chemical behaviour of the anode mixture. On the other hand, the baking index was used to estimate the shrinking index, which includes the effect of the pitch pyrolysis reaction on modeling the baking process.

Secondly, the thermal expansion coefficient was calculated using the cooling period of the strain in the dilatometry such that the thermal and chemical strains were obtained. The analysis of results indicated that the chemical strain due to the pitch pyrolysis is a superimposition of the strain due to the increase in the internal gas pressure caused by the devolatilization process and the strain due to the chemical shrinkage of the anode caused by the pitch carbonization. Between 148 and 636 °C, the chemical strain due to pitch pyrolysis mainly causes the volume expansion of the anode through the internal gas pressure, while above 636 °C, the chemical strain due to pitch pyrolysis dominates in shrinking the anode volume through the chemical shrinkage of the pitch binder due to the pitch carbonization.

Thirdly, the creep behaviour of the anode at different high temperatures were characterized. The analysis of the obtained results implied that the anode creep was mainly impacted by the internal gas pressure due to the devolatilization process, the pitch viscosity change due to the phase transformation, and the compressive strength of the anode. Parameters involved in the Burger’s rheological model were identified using the results of creep tests. Using these parameters, viscoelastic and viscoplastic strains of the anode in the axial direction were predicted at different baking temperatures. Several relevant parameters were further expressed as a function of the shrinking index to take the chemo-mechanical coupling effect into account. They provide a tool to investigate the chemo-mechanical coupling effect of the anode mixture.

This work highlighted some relevant insights into the thermo-chemo-mechanical properties of the anode as well as phenomena such as the expansion due to the increase in the internal gas pressure and the chemical shrinkage of the anode during the baking process. These findings pave the way for modeling the thermo-chemo-poromechanical behaviour of the anode during the baking process. The obtained properties could facilitate the development of computational tools, which would regulate fundamental guidelines for better controlling and optimization of the baking process in the anode production industry.

## Figures and Tables

**Table 1:** Size distribution of coke aggregates in anode samples [28].

Size Range (US mesh)	Mass (wt %)
−4 + 8	22.0
−8 + 14	10.0
−14 + 30	11.5
−30 + 50	12.7
−50 + 100	8.8
−100 + 200	10.8
>−200	24.2

**Table 2:** Sample dimensions for different characterizations.

Characterizations	Type	Diameter × Height (mm × mm)
Thermogravimetric analysis	Green	50 × 50
Dilatometry		50 × 50
Creep test	Baked	50 × 100

**Table 3:** Parameters in the Burger’s rheological model for different baking temperatures.

	Parameter	$E_{\chi }^{\mathrm{av}}$ (Pa · s)	$\nu_{\chi }^{\mathrm{av}}$	$E_{\chi }^{\mathrm{ae}}$ (Pa)	$\nu_{\chi }^{\mathrm{ae}}$	$E_{\chi }^{v}$ (Pa · s )	$\nu_{\chi }^{v}$
Temperature							
**200 °C**		5.000 × 10^10^	0.375	4.177 × 10^7^	0.375	3.012 × 10^13^	0.293
**300 °C**		1.000 × 10^11^	0.375	3.217 × 10^7^	0.375	1.279 × 10^13^	0.380
**400 °C**		4.893 × 10^11^	0.450	8.576 × 10^7^	0.301	1.000 × 10^13^	0.200
**500 °C**		3.457 × 10^12^	0.101	2.158 × 10^8^	0.365	1.577 × 10^13^	0.383
**600 °C**		3.208 × 10^12^	0.301	2.146 × 10^8^	0.299	7.831 × 10^12^	0.299
**700 °C**		1.503 × 10^12^	0.420	1.051 × 10^8^	0.358	8.664 × 10^12^	0.199

## Data Availability

Not applicable.

## Appendix A. Sensitivity Analysis of the Inverse Identification of Parameters

This appendix aims at investigating the sensitivity of the inverse identification procedure used in the Section 4.4 to the Poisson-like parameters $\nu_{\chi }^{\mathrm{av}}$, $\nu_{\chi }^{\mathrm{ae}}$ and $\nu_{\chi }^{v}$ involved in the Burger’s rheological model (Equations (22) and (23)). Since the radial strains of creep tests were not measured, it is expected that the minimization of the objective function in the Equation (31) will be less sensitive to these parameters. Without loss of generality, let us consider the results of the creep test at 400 °C (Figure 17). Poisson-like parameters $\nu_{\chi }^{\mathrm{av}}$, $\nu_{\chi }^{\mathrm{ae}}$ and $\nu_{\chi }^{v}$ are assumed belonging to the interval [0, 0.5] which will be discretized into n sub-intervals $I_{i}=\left[x_{i},x_{i+1}\right]$ such that:

(A1) $$\overset{n}{\underset{i=1}{\cup }}\left[x_{i},x_{i+1}\right]=\left[0,0.5\right]$$

In this appendix, the inverse identification of the material’s parameters involved in the Equation (23) is carried out by constraining the Poisson-like parameters $\nu_{\chi }^{\mathrm{av}}$, $\nu_{\chi }^{\mathrm{ae}}$ and $\nu_{\chi }^{v}$ to belong to several sub-intervals $\left[x_{i},x_{i+1}\right]$ such that the total number of possible combinations for these 3 parameters is n^3^. Table A1, Table A2 and Table A3 summarize the results obtained for all parameters by considering cases corresponding to n = 2 (8 combinations), n = 3 (27 combinations) and n = 4 (64 combinations). Notice that all optimal sets of parameters allow the Burger’s rheological model to predict the experimental trends with a good agreement. Moreover, these results show that the minimization of the objective function leads to almost close results for the Young-like Modulus parameters $E_{\chi }^{\mathrm{av}}$, $E_{\chi }^{\mathrm{ae}}$ and $E_{\chi }^{v}$, regardless of the constraints imposed on the variation of the Poisson-like parameters.

**Table A1:** Inverse identification results for the case n = 2 ($I_{1}=\left[0.005,0.25\right]$ and $I_{2}=\left[0.25,0.5\right]$ ).

$E_{\chi }^{\mathrm{av}}$	$\nu_{\chi }^{\mathrm{av}}$	$E_{\chi }^{\mathrm{ae}}$	$\nu_{\chi }^{\mathrm{ae}}$	$E_{\chi }^{v}$	$\nu_{\chi }^{v}$
5.220 × 10^11^	0.300	8.716 × 10^7^	0.200	1.000 × 10^13^	0.200
5.347 × 10^11^	0.300	8.519 × 10^7^	0.200	1.000 × 10^13^	0.301
4.702 × 10^11^	0.200	8.709 × 10^7^	0.435	1.000 × 10^13^	0.200
4.818 × 10^11^	0.200	8.512 × 10^7^	0.435	1.000 × 10^13^	0.301
4.626 × 10^11^	0.449	8.196 × 10^7^	0.203	1.033 × 10^13^	0.203
4.662 × 10^11^	0.449	8.119 × 10^7^	0.201	1.000 × 10^13^	0.301
4.893 × 10^11^	0.450	8.576 × 10^7^	0.301	1.000 × 10^13^	0.200
5.026 × 10^11^	0.450	8.374 × 10^7^	0.301	1.000 × 10^13^	0.301

**Table A2:** Inverse identification results for the case n = 3 ($I_{1}=\left[0.05,0.17\right]$, $I_{2}=\left[0.17,0.34\right]$ and $I_{3}=\left[0.34,0.5\right]$.

$E_{\chi }^{\mathrm{av}}$	$\nu_{\chi }^{\mathrm{av}}$	$E_{\chi }^{\mathrm{ae}}$	$\nu_{\chi }^{\mathrm{ae}}$	$E_{\chi }^{v}$	$\nu_{\chi }^{v}$
5.020 × 10^11^	0.170	8.866 × 10^7^	0.005	1.000 × 10^13^	0.005
5.096 × 10^11^	0.170	8.748 × 10^7^	0.005	1.000 × 10^13^	0.170
5.314 × 10^11^	0.170	8.409 × 10^7^	0.005	1.000 × 10^13^	0.341
4.438 × 10^11^	0.005	8.852 × 10^7^	0.333	1.000 × 10^13^	0.009
4.488 × 10^11^	0.005	8.731 × 10^7^	0.337	1.000 × 10^13^	0.173
4.684 × 10^11^	0.005	8.391 × 10^7^	0.339	1.000 × 10^13^	0.342
4.157 × 10^11^	0.005	8.852 × 10^7^	0.389	1.000 × 10^13^	0.005
4.218 × 10^11^	0.005	8.735 × 10^7^	0.389	1.000 × 10^13^	0.171
4.409 × 10^11^	0.005	8.392 × 10^7^	0.388	1.000 × 10^13^	0.342
4.489 × 10^11^	0.318	8.534 × 10^7^	0.005	1.054 × 10^13^	0.009
4.489 × 10^11^	0.318	8.534 × 10^7^	0.005	1.015 × 10^13^	0.193
4.614 × 10^11^	0.333	8.151 × 10^7^	0.005	1.003 × 10^13^	0.357
4.923 × 10^11^	0.337	8.845 × 10^7^	0.173	1.000 × 10^13^	0.005
4.966 × 10^11^	0.340	8.720 × 10^7^	0.170	1.000 × 10^13^	0.170
5.189 × 10^11^	0.340	8.377 × 10^7^	0.170	1.000 × 10^13^	0.341
4.553 × 10^11^	0.173	8.868 × 10^7^	0.428	1.000 × 10^13^	0.005
4.608 × 10^11^	0.170	8.751 × 10^7^	0.428	1.000 × 10^13^	0.170
4.808 × 10^11^	0.170	8.411 × 10^7^	0.427	1.000 × 10^13^	0.341
4.312 × 10^11^	0.420	7.708 × 10^7^	0.048	1.193 × 10^13^	0.005
4.565 × 10^11^	0.420	8.416 × 10^7^	0.170	1.017 × 10^13^	0.193
4.726 × 10^11^	0.422	8.167 × 10^7^	0.170	1.000 × 10^13^	0.342
4.636 × 10^11^	0.420	8.507 × 10^7^	0.193	1.043 × 10^13^	0.005
4.543 × 10^11^	0.441	8.140 × 10^7^	0.170	1.058 × 10^13^	0.171
4.667 × 10^11^	0.439	8.009 × 10^7^	0.170	1.005 × 10^13^	0.342
5.010 × 10^11^	0.479	8.714 × 10^7^	0.389	1.005 × 10^13^	0.005
4.943 × 10^11^	0.464	8.625 × 10^7^	0.341	1.000 × 10^13^	0.170
5.153 × 10^11^	0.469	8.241 × 10^7^	0.341	1.000 × 10^13^	0.341

**Table A3:** Inverse identification results for the case n = 4 ($I_{1}=\left[0.005,0.125\right]$, $I_{2}=\left[0.125,0.25\right]$, $I_{3}=\left[0.25,0.375\right]$ and $I_{4}=\left[0.375,0.5\right]$ ).

$E_{\chi }^{\mathrm{av}}$	$\nu_{\chi }^{\mathrm{av}}$	$E_{\chi }^{\mathrm{ae}}$	$\nu_{\chi }^{\mathrm{ae}}$	$E_{\chi }^{e}$	$\nu_{\chi }^{v}$
5.125 × 10^11^	0.125	8.880 × 10^7^	0.005	1.000 × 10^13^	0.006
5.165 × 10^11^	0.125	8.817 × 10^7^	0.005	1.000 × 10^13^	0.125
5.282 × 10^11^	0.125	8.630 × 10^7^	0.005	1.000 × 10^13^	0.250
5.484 × 10^11^	0.125	8.328 × 10^7^	0.005	1.000 × 10^13^	0.376
4.806 × 10^11^	0.010	8.863 × 10^7^	0.250	1.000 × 10^13^	0.010
4.843 × 10^11^	0.010	8.800 × 10^7^	0.250	1.000 × 10^13^	0.125
4.957×1 0^11^	0.006	8.613 × 10^7^	0.250	1.000 × 10^13^	0.250
5.137 × 10^11^	0.006	8.308 × 10^7^	0.250	1.000 × 10^13^	0.376
4.233 × 10^11^	0.006	8.852 × 10^7^	0.375	1.000 × 10^13^	0.006
4.263 × 10^11^	0.005	8.788 × 10^7^	0.375	1.000 × 10^13^	0.125
4.364 × 10^11^	0.005	8.601 × 10^7^	0.375	1.000 × 10^13^	0.250
4.538 × 10^11^	0.006	8.297 × 10^7^	0.375	1.000 × 10^13^	0.376
4.065 × 10^11^	0.005	8.853 × 10^7^	0.406	1.000 × 10^13^	0.020
4.144 × 10^11^	0.018	8.791 × 10^7^	0.406	1.000 × 10^13^	0.125
4.300 × 10^11^	0.010	8.603 × 10^7^	0.390	1.000 × 10^13^	0.250
4.464 × 10^11^	0.005	8.292 × 10^7^	0.388	1.000 × 10^13^	0.376
4.771 × 10^11^	0.250	8.828 × 10^7^	0.018	1.000 × 10^13^	0.018
4.810× 10^11^	0.250	8.765 × 10^7^	0.018	1.000 × 10^13^	0.125
4.887 × 10^11^	0.250	8.570 × 10^7^	0.006	1.000 × 10^13^	0.250
5.087 × 10^11^	0.250	8.262 × 10^7^	0.005	1.000 × 10^13^	0.376
5.083 × 10^11^	0.250	8.873 × 10^7^	0.125	1.000 × 10^13^	0.006
5.122 × 10^11^	0.250	8.810 × 10^7^	0.125	1.000 × 10^13^	0.125
5.241 × 10^11^	0.250	8.623 × 10^7^	0.125	1.000 × 10^13^	0.250
5.439 × 10^11^	0.250	8.322 × 10^7^	0.125	1.000 × 10^13^	0.375
4.657 × 10^11^	0.125	8.864 × 10^7^	0.375	1.000 × 10^13^	0.006
4.694 × 10^11^	0.125	8.801 × 10^7^	0.375	1.000 × 10^13^	0.125
4.804 × 10^11^	0.125	8.614 × 10^7^	0.375	1.000 × 10^13^	0.250
4.988 × 10^11^	0.125	8.310 × 10^7^	0.375	1.000 × 10^13^	0.376
4.419 × 10^11^	0.125	8.863 × 10^7^	0.421	1.000 × 10^13^	0.010
4.453 × 10^11^	0.125	8.801 × 10^7^	0.421	1.000 × 10^13^	0.125
4.582 × 10^11^	0.125	8.614 × 10^7^	0.417	1.000 × 10^13^	0.250
4.760 × 10^11^	0.125	8.305 × 10^7^	0.417	1.000 × 10^13^	0.376
4.263 × 10^11^	0.375	8.075 × 10^7^	0.006	1.141 × 10^13^	0.006
4.532 × 10^11^	0.313	8.625 × 10^7^	0.018	1.019 × 10^13^	0.125
4.278 × 10^11^	0.352	8.423 × 10^7^	0.010	1.000 × 10^13^	0.250
4.321 × 10^11^	0.375	7.973 × 10^7^	0.006	1.000 × 10^13^	0.390
4.509 × 10^11^	0.375	8.741 × 10^7^	0.125	1.000 × 10^13^	0.034
4.577 × 10^11^	0.375	8.634 × 10^7^	0.125	1.000 × 10^13^	0.164
4.693 × 10^11^	0.375	8.454 × 10^7^	0.125	1.000 × 10^13^	0.266
4.878 × 10^11^	0.375	8.175 × 10^7^	0.125	1.000 × 10^13^	0.375
5.002 × 10^11^	0.375	8.854 × 10^7^	0.250	1.000 × 10^13^	0.010
5.042 × 10^11^	0.375	8.790 × 10^7^	0.250	1.000 × 10^13^	0.125
5.161 × 10^11^	0.375	8.603 × 10^7^	0.250	1.000 × 10^13^	0.250
5.360 × 10^11^	0.375	8.301 × 10^7^	0.250	1.000 × 10^13^	0.375
4.726 × 10^11^	0.250	8.874 × 10^7^	0.445	1.000 × 10^13^	0.006
4.754 × 10^11^	0.250	8.811 × 10^7^	0.446	1.000 × 10^13^	0.125
4.864 × 10^11^	0.250	8.625 × 10^7^	0.446	1.000 × 10^13^	0.250
5.056 × 10^11^	0.250	8.322 × 10^7^	0.445	1.000 × 10^13^	0.376
4.403 × 10^11^	0.437	7.347 × 10^7^	0.045	1.242 × 10^13^	0.005
4.440 × 10^11^	0.437	7.721 × 10^7^	0.096	1.149 × 10^13^	0.125
4.476 × 10^11^	0.437	7.872 × 10^7^	0.120	1.070 × 10^13^	0.250
4.335 × 10^11^	0.437	7.102 × 10^7^	0.006	1.050 × 10^13^	0.429
4.485 × 10^11^	0.437	7.904 × 10^7^	0.125	1.128 × 10^13^	0.085
4.592 × 10^11^	0.437	8.285 × 10^7^	0.188	1.056 × 10^13^	0.125
4.864 × 10^11^	0.437	8.420 × 10^7^	0.250	1.001 × 10^13^	0.250
4.984 × 10^11^	0.447	8.076 × 10^7^	0.250	1.000 × 10^13^	0.376
4.704 × 10^11^	0.437	8.632 × 10^7^	0.250	1.017 × 10^13^	0.018
4.695 × 10^11^	0.437	8.644 × 10^7^	0.250	1.000 × 10^13^	0.125
4.807 × 10^11^	0.441	8.436 × 10^7^	0.250	1.000 × 10^13^	0.250
4.986 ×10^11^	0.447	8.076 × 10^7^	0.250	1.000 × 10^13^	0.376
4.987 × 10^11^	0.476	8.686 × 10^7^	0.378	1.000 × 10^13^	0.125
5.020 × 10^11^	0.472	8.720 × 10^7^	0.382	1.000 × 10^13^	0.125
5.095 × 10^11^	0.476	8.508 × 10^7^	0.378	1.000 × 10^13^	0.250
5.301 × 10^11^	0.477	8.175 × 10^7^	0.375	1.000 × 10^13^	0.375

## Appendix B. A Summary of Parameters Involved in the Mathematical Model

a	slope of the model of the baking index between 527 and 1000 °C
$a_{h}$	heating rate
b	intercept of the model of the baking index between 527 and 1000 °C
$E_{a}$	activation energy
$k_{o}$	pre-exponential factor
R	universal gas constant
$\alpha_{T}$	thermal expansion coefficient
$\lambda_{1}$, $\lambda_{2}$	coefficients related to the relaxation time corresponding to the viscoelastic behaviour of the material
$\left(E_{\chi }^{e},\nu_{\chi }^{e}\right)$	Young Modulus and the Poisson’s ratio that describe the elastic behaviour with respect to the shrinking index
$\left(E_{\chi }^{\mathrm{ae}},v_{\chi }^{\mathrm{ae}}\right)$	elastic moduli characterizing the viscoelastic behaviour with respect to the shrinking index
$\left(E_{\chi }^{\mathrm{av}},\nu_{\chi }^{\mathrm{av}}\right)$	viscous moduli characterizing the viscoelastic behaviour with respect to the shrinking index
$\left(E_{\chi }^{v},\nu_{\chi }^{v}\right)$	viscous moduli characterizing the viscoplastic behaviour with respect to the shrinking index
${\left(K_{\chi }^{a}\right)}^{-1}$, ${\left(G_{\chi }^{a}\right)}^{-1}$	constants related to hydrostatic and deviatoric viscoelastic behaviour with respect to the shrinking index
